# The circadian rhythms regulated by Cx43-signaling in the pathogenesis of Neuromyelitis Optica

**DOI:** 10.3389/fimmu.2022.1021703

**Published:** 2023-01-16

**Authors:** Huiru Xue, Minghui Wu, Yongle Wang, Yunfei Zhao, Meini Zhang, Hui Zhang

**Affiliations:** ^1^ Department of Neurology, First Hospital of Shanxi Medical University, Taiyuan, Shanxi, China; ^2^ First Clinical Medical College of Shanxi Medical University, Taiyuan, Shanxi, China; ^3^ Department of Medical Imaging, First Hospital of Shanxi Medical University, Taiyuan, Shanxi, China

**Keywords:** Neuromyelitis Optica (NMO), circadian rhythms, astrocyte, Cx43, Bmal1

## Abstract

**Introduction:**

Neuromyelitis Optica (NMO) is an inflammatory demyelinating disease of the central nervous system (CNS). NMO manifests as selective and severe attacks on axons and myelin of the optic nerve and spinal cord, resulting in necrotic cavities. The circadian rhythms are well demonstrated to profoundly impact cellular function, behavior, and disease. This study is aimed to explore the role and molecular basis of circadian rhythms in NMO.

**Methods:**

We used an Aquaporin 4(AQP4) IgG-induced NMO cell model in isolated astrocytes. The expression of Cx43 and Bmal1 were detected by real-time PCR and Western Blot. TAT-Gap19 and DQP-1105 were used to inhibit Cx43 and glutamate receptor respectively. The knockdown of Bmal1 were performed with the shRNA containing adenovirus. The levels of glutamate, anterior visual pathway (AVP), and vasoactive intestinal peptide (VIP) were quantified by ELISA kits.

**Results:**

We found that Bmal1 and Clock, two essential components of the circadian clock, were significantly decreased in NMO astrocytes, which were reversed by Cx43 activation (linoleic acid) or glutamate. Moreover, the expression levels of Bmal1 and Clock were also decreased by Cx43 blockade (TAT-Gap19) or glutamate receptor inhibition (DQP-1105). Furthermore, adenovirus-mediated Bmal1 knockdown by shRNA (Ad-sh-Bmal1) dramatically decreased the levels of glutamate, AVP, and VIP from neurons, and significantly down-regulated the protein level of Cx43 in NMO astrocytes with Cx43 activation (linoleic acid) or glutamate treatment. However, Bmal1 knockdown did not alter these levels in normal astrocytes with Cx43 blockade (TAT-Gap19) or glutamate receptor inhibition (DQP-1105).

**Discussion:**

Collectively, these results suggest that Cx43-glutamate signaling would be a critical upstream regulator that contributes to the NMO-induced rhythmic damage in SCN astrocytes.

## 1 Introduction

The rare disease Neuromyelitis Optica (NMO) causes debilitating, occasionally fatal changes in the central nervous system (CNS) including attacks of severe blindness and paralysis (1, 2). It is caused by antibodies directed against the water channel protein aquaporin-4 (AQP4) that is concentrated at the blood-brain barrier in astrocytic foot processes (3–5). An AQP4-specific antibody (NMO-IgG) is a B cell-dependent antibody subclass, emphasizing the synergistic role played by cellular and humoral arms of adaptive immunity during the pathogenesis of NMO (6, 7). In 2015, the International NMO Diagnostic Team developed a new diagnostic standard for NMO spectrum disorders (NMOSD), which further stratified the diagnosis of NMOSD into AQP-4-IgG-positive and -negative (8).

Circadian rhythms govern the periodicity of physiological processes in living beings (9). A critical role is played by it in animal behavioral and physiological processes as well as disease states (10–12). A clock gene activity network controls this rhythm, which is affected by environmental cycles (especially light) (13). There has been research on the influence and importance of the circadian clock for some autoimmune and inflammatory conditions, including rheumatoid arthritis, and inflammatory bowel disease (14–16). In NMOSD patients, sleep disturbances are also associated with fatigue, according to a recent clinical study (17). Although these reports have not been investigated in-depth, they suggest that changes in daily rhythms may be associated with the disease course of NMOSD (18). The main clinical manifestations of NMOSD are visual loss and paralysis, as well as an obvious circadian rhythm disorder (8). Conversely, the disorder of circadian rhythm can induce the disorder of immune function and increase the risk of disease recurrence (19). Therefore, the regulatory role of circadian rhythm may largely contribute to disease prevention and treatment of NMOSD.

There have been many cases reported that NMOSD patients with hypothalamic impairment to present with symptoms of sleep rhythm disorder (17). Sleep has the function of consolidating immune memory and enhancing immune defense (20, 21). And the immune status of animals and humans, such as cytokines and other immune mediators, will also affect the punctuality process of the circadian rhythm (22–24). Therefore, there is a bidirectional relationship between the circadian system and the immune system (25). The Suprachiasmatic Nucleus (SCN), known as the “master clock” in mammals, receives light-entrained signals through the retinal hypothalamus bundle to keep the body adapted to its internal and external environment (26–28). NMOSD lesions are most easily involved in the optic nerve, optic chiasma, and hypothalamus where AQP4 is highly expressed (4). The same anatomical basis may be an important site for the bidirectional influence of NMO and the circadian system (29, 30). So far, the research on the characteristics of NMOSD circadian rhythm disorder is not in-depth enough, and the interaction between NMOSD and circadian rhythm disorder and its mechanism is still not clear.

**Figure 1:**
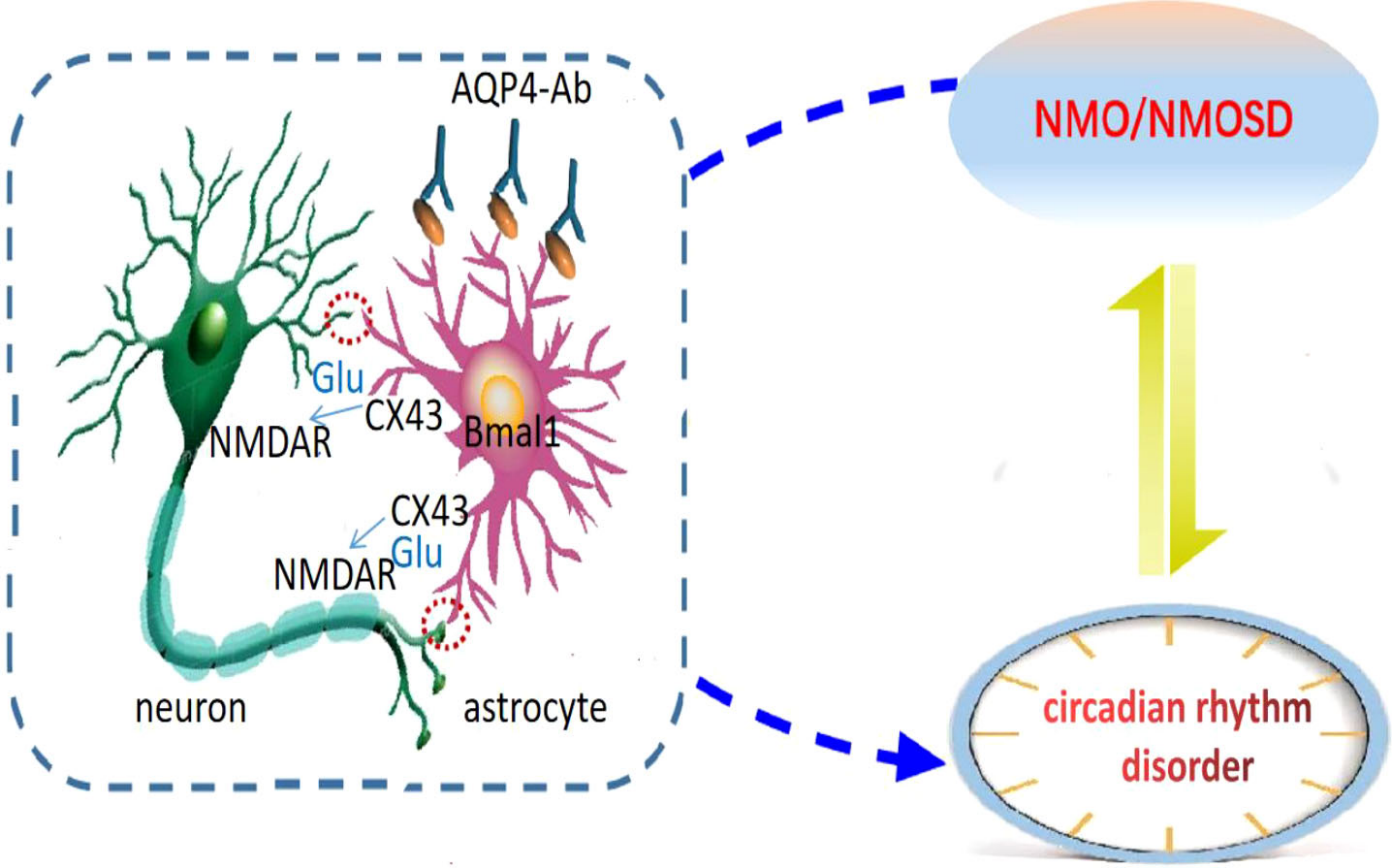
Astrocytic regulation hypothesis of circadian disruption in NMO. Neurons are the main pacing cells in SCN, which integrate and synchronize the circadian rhythm of each cell through the neuronal network. Astrocytes are involved in the pathogenesis of NMOSD, and are also important regulators of circadian rhythm. They affect circadian rhythm through glutamate pathway. We hypothesized that the expression of clock gene Bmal1 in NMO astrocytes would affect Cx43 and glutamate levels, and then regulate the release of neuronal synchronization signals. Circadian system and immune system interact in a bidirectional manner: NMO disease state can lead to circadian rhythm disorder, and circadian rhythm disorder can also induce NMO disease recurrence.

Prolo et al. confirmed the existence of clock genes expressed rhythmically in astrocytes (31), and Brancaccio et al. reported that astrocytes autonomously initiate and maintain complex diurnal behavior of mammals through glutamate energy signals (32). It was found that inhibition of Connexin 43 (Cx43) specifically expressed in the astrocytes could interfere with the release of glutamate to affect the diurnal oscillation of clock gene expression in the astrocytes, and the increase of Cx43 expression could induce the formation of the clock system (33). Cx43-mediated gap junction supports neuron-glial interaction (34). This suggests that the astrocytes are important regulators of circadian rhythms (35). The target antigen of the AQP4 antibody is AQP4 located on the astrocyte’s foot process, and the astrocytes are involved in the pathogenesis of NMOSD (36–38). The astrocytes with dual identities are likely to be the hubs of bidirectional regulation of NMOSD and circadian rhythm disorder, but their roles and mechanisms in NMOSD circadian rhythm disorder are still unknown. Cx43-glutamate may play an important regulatory role in astrocytes’ participation in circadian rhythm, but no relevant reports have been reported in NMOSD and further studies are needed (**Figure 1**).

In conclusion, it is of great theoretical and practical clinical significance to explore the interaction between NMOSD medium astrocytes and circadian rhythm regulation and its underlying mechanism. In this study, anti-AQP4 IgG was used to induce an NMO cell model to evaluate the potential role of Cx43 on the NMO-induced neuron injury and circadian rhythms damage. We found that the Cx43-glutamate signaling and circadian rhythm regulating genes (Bmal1 and Clock) were significantly decreased in a time-dependent manner. Moreover, we found that the levels of Bmal1 and Clock were significantly decreased in normal astrocytes with a specific Cx43 hemichannel (Cx43 HC) inhibitor TAT-Gap19. Conversely, they were increased in the NMO-induced astrocytes with a Cx43 activator LA. Our results also suggested that the decreasing of Bmal1 contributes to the disease aggressiveness of NMO. Activation of Cx43-glutamate signaling would be promising strategy for the therapy of NMOSD and prevent NMO-damaged circadian rhythms.

## 2 Materials and methods

### 2.1 Primary astrocytes isolation and culture

Mixed SCN was removed from mice and minced with scissors in ice-cold HBSS (Gibco), then digested with 0.25% trypsin solution (Gibco, Carlsbad, CA, USA) at 37°C for 30 min. The dissociated cells were rinsed and re-suspended in high glucose DMEM and counted. Cells were plated in a T25 flask at a density of 30000 cells/cm^2^. After 2 days, the high glucose DMEM medium containing 10% FBS (Gibco) and 1% Penicillin/Streptomycin (Gibco) was changed to remove cell debris. 9–10 days later, we passaged the first split astrocyte population at the appropriate cell concentration for the experiment. The purity of astrocytes was confirmed by immunofluorescence and fluorescence activated cell sorting (FACS) analysis of GFAP-positive cells, which was up to 99.8%. Briefly, the cells were stained with FITC labeled anti-GFAP antibody (Biolegend, USA). Data were collected using a FACSCalibur cytometer (BD Biosciences, San Jose, CA) and analyzed using FlowJo (Tree Star) software. For the NMO induction of astrocytes, the anti-AQP4 IgG (5 ug/mL) and human complement (10 ug/mL) was added into the culture medium of astrocytes for the indicated time.

### 2.2 Primary neurons isolation and culture

Cortical tissues were removed from mice mechanically isolated in an Opti MEM medium (Gibco). The cells were cultured on a flask coated with 0.05 mg/mL poly-D-lysine in neurobasal medium supplemented with B27 (Gibco) at 37°C and 5%CO_2_, and the medium was changed every 3 days. After 12-15 days, after fixation with 4% paraformaldehyde, the concentration and purity of neurons were confirmed by staining with MAP2 antibody. To evaluate the effect of astrocytes on neurons, the cultured astrocytes on the bottom (1.5×10^5^ cells/well in 6-well plates) and the cultured neurons in the Transwell chamber (5×10^4^ cells/well in 6-well Transwell plates, 0.4 μm) were co-cultured for 24 hours to observe the effect of astrocytes on neurons.

### 2.3 NMOSD *in vitro* astrocyte model

The NMOSD *in vitro* astrocyte model was established as previously reported (39). Briefly, the anti-AQP4 IgG (5 μg/mL) and human complement (C3,10 μg/mL, Complement Technology, Beijing) were added into the culture medium of astrocytes for the indicated time. For the co-culture of astrocytes and neurons, the astrocytes were seeded in the lower chamber of Transwell (Corning, USA), the neurons were seeded in the upper chamber of Transwell (Corning, USA).

### 2.4 Quantitative real-time PCR (qRT-PCR)

Total RNA was lysed with TRIzol reagent and cDNA was synthesized using a one-step RT-PCR kit (Thermo Fisher Scientific). Real-time fluorescence quantitative PCR (qRT-PCR) was performed using the ABI Vii7 system (Applied Biosystems, USA). GAPDH is used as a housekeeping gene. The 2^-△△CT^ cycle threshold method (40) was used to calculate relative gene expression levels. Primers used for QRT-PCR analysis are listed in **Table 1**.

**Table 1:** Primer sequences for QRT-PCR analysis.

**Gene**	**Forward primer (5’-3’)**	**Reverse primer (5’-3’)**
Clock	CCTATCCTACCrrCGCCACACA	TCCCGTGGAGCAACCTAGAT
Bmal1	TCGTTGCAATCGGGCG	CCGTATTTCCCCGTTCGC
GAPDH	AAATGGTGAAGGTCGGTGTGAAC	CAACAATCTCCACTTTGCCACTG

### 2.5 Enzyme-linked immunosorbent assay (ELISA) assay

Levels of Glu, ANP, and VIP in the cell culture medium were determined using commercially available ELISA kits (eBioscience Co., San Diego, CA, USA) according to the manufacturer’s instructions. In simple terms, take 100 μL supernatant, diluted standard, quality control, and diluted buffer (blank) and place on a pre-coated plate containing monoclonal antibody for 2 hours. Add 100 μL biotin-labeled antibody and incubate for 1 hour. Wash the plate, add 100 μL streptavidin-HRP conjugate, and incubate in the dark for 30 min. Adding 100 μL substrate and stop solution indicates the last steps before reading the absorbance (450nm) on the microplate reader.

### 2.6 Western blot

Total cellular proteins were lysed by RIPA buffer containing protease inhibitors (Beyotime, China). The protein extractions were harvested and quantified by bicinchoninic acid (BCA) analysis (Beyotime, China). Protein extractions were separated by 10% SDS-PAGE and transferred onto polyvinylidene fluoride (PVDF) membranes (Millipore, USA). The membranes were incubated with antibodies against Cx43 (Abcam, Cambridge, MA, USA) and GAPDH (Abcam, Cambridge, MA, USA) as previously described. Using GAPDH as endogenous controls, we determined the loading of total proteins.

### 2.7 Statistical analysis

Data are presented as mean ± SD (mean ± standard deviation) for at least three independent experiments. Statistical analyses have been performed using GraphPad Prism 9 software (GraphPad Software Inc., La Jolla, CA, USA). One-way ANOVA and Student t-tests were used to compare means between groups. It is statistically significant when *P* less than 0.05 is used.

## 3 Results

### 3.1 The expression of Cx43 protein and circadian rhythm regulating gene (Bmal1 and Clock) are time-dependently decreased in astrocytes after NMO induction

**Figure 2:**
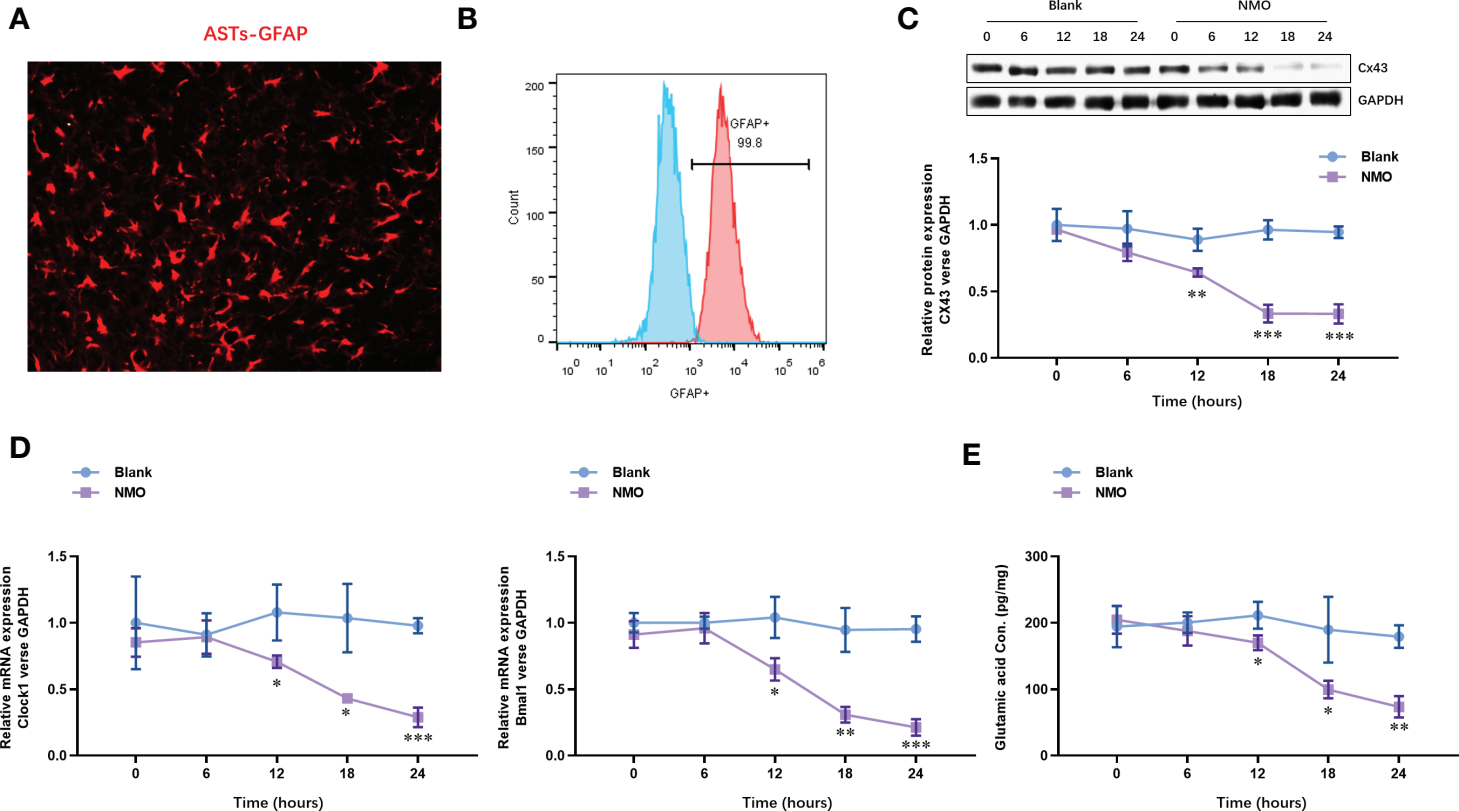
The expression of Cx43 protein and circadian rhythm regulating gene (Clock and Bmal1) are time-dependently decreased in astrocytes after NMO induction. **(A)** The identity of isolated SCN astrocytes was confirmed with GFAP by immunofluorescence staining. **(B)** The purity of SCN astrocytes was further confirmed by fluorescence activated cell sorting (FACS) analysis. **(C)** The protein levels of Cx43 in SCN astrocytes with or without NMO induction were analyzed by Western Blot. **(D)** The mRNA levels of circadian rhythm regulating genes (Clock and Bmal1) in SCN astrocytes with or without NMO were analyzed by real-time PCR. **(E)** the levels of Glu in SCN astrocytes with or without NMO-IgG induction were analyzed by ELISA. *P<0.05, **P<0.01, ***P<0.001, compared with the blank group. N=3.

To understand the effect of NMO induction on the level of Cx43 and circadian rhythm-regulating genes (Bmal1 and Clock), we constructed an anti-AQP4 IgG-induced NMO model in SCN astrocytes. The SCN astrocytes were isolated from the SCN tissues. The identity of SCN astrocytes was confirmed with the expression of an astrocyte-specific marker, GFAP, by immunofluorescence staining (**Figure 2A**). The purity of SCN astrocytes was further confirmed by fluorescence activated cell sorting (FACS) analysis, which was up to 99.8% (**Figure 2B**). The isolated astrocytes were then treated with anti-AQP4 IgG (5 μg/ml) or control IgG (5 μg/ml) and human complement (10 μg/ml) for 0,6,12,18 and 24 hours. The protein level of Cx43 was significantly decreased after 12 hours of treatment with NMO induction (**Figure 2C**). Moreover, the mRNA levels of circadian rhythm-regulating genes (Bmal1 and Clock) were decreased after 12 hours of treatment with NMO induction (**Figure 2D**). Considering neurotransmitter glutamate (Glu) is mainly taken up by surrounding astrocytes after interaction with receptors in the synapse, we then detected the levels of Glu in astrocytes. Notably, the level of Glu also dramatically decreased after 12 hours of treatment with NMO induction (**Figure 2E**). These results suggested that the level of Cx43 protein and circadian rhythm regulating gene (Bmal1and Clock) may be involved in the NMO induction in astrocytes.

### 3.2 Astrocytes after NMO induction significantly decreased the neuropeptides (AVP and VIP) released from co-cultured neurons

**Figure 3:**
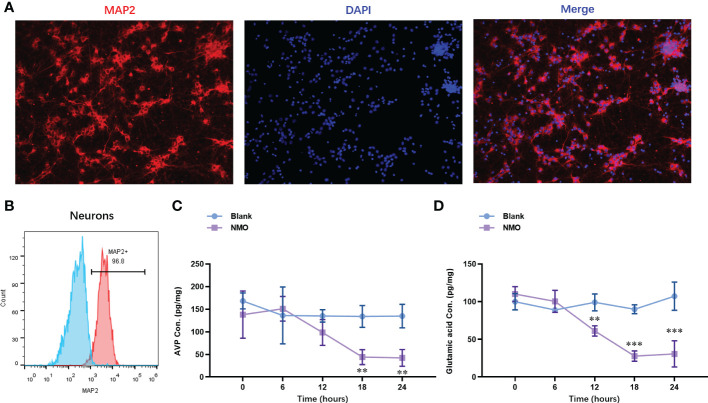
Astrocytes after NMO induction significantly decreased the neuropeptides (AVP and VIP) released from co-cultured neurons. **(A)** The identity of neurons was confirmed with the expression of MAP2 by immunofluorescence staining. **(B)** The purity of neurons was further confirmed by fluorescence activated cell sorting (FACS) analysis, which was up to 96.8%. After the co-culture of NMO inducted astrocytes and neurons for 0,6,12,18 and 24 hours. The protein level of AVP **(C)** and VIP **(D)** were detected by ELISA kits. **P<0.01, ***P<0.001, compared with the blank group. N=3.

To evaluate the NMO astrocytes-induced neuron circadian rhythm dysregulation, we constructed a co-culture model of astrocytes and neurons. The neurons were isolated from the cortical tissues. The identity of neurons was confirmed with the expression of a neuron-specific marker, MAP2, by immunofluorescence staining (**Figure 3A**). The purity of neurons was further confirmed by fluorescence activated cell sorting (FACS) analysis, which was up to 96.8% (**Figure 3B**). After the co-culture of NMO inducted astrocytes and neurons were for 0,6,12,18 and 24 hours. The protein level of neuropeptides, including arginine vasopressin (AVP) and vasoactive intestinal polypeptide (VIP), were detected by ELISA kits. The level of AVP was decreased at 24 hours of co-culture with NMO astrocytes (**Figure 3C**), whereas VIP was significantly decreased after 12 hours of treatment with co-culture with NMO astrocytes (**Figure 3D**). These results demonstrated an obvious NMO astrocytes-induced AVP and VIP circadian rhythm release from the neuron.

### 3.3 Cx43-glutamate signaling inhibition contributes to the NMO-induced circadian rhythm dysregulation

**Figure 4:**
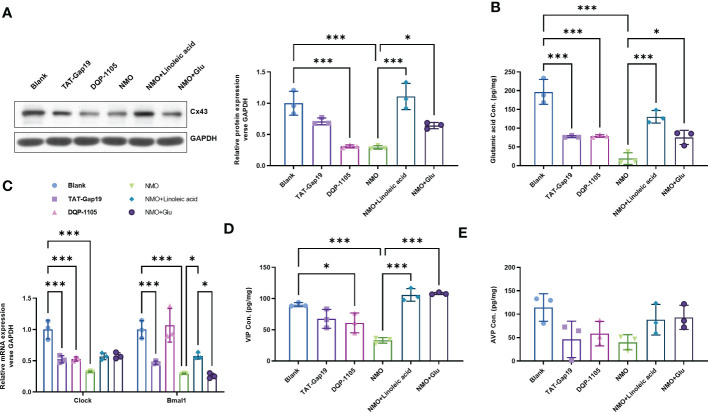
Cx43-glutamate signaling inhibition contributes to the NMO-induced circadian rhythm dysregulation. **(A)** The protein level of Cx43 in normal astrocytes with or without TAT-Gap19/DQP-1105 or NMO astrocytes with or without LA/glutamate were analyzed by Western Blot. **(B)** The glutamate levels in normal astrocytes with or without TAT-Gap19/DQP-1105 or NMO astrocytes with or without LA/glutamate were analyzed by ELISA kit. **(C)** The mRNA levels of Clock and Bmal1 in normal astrocytes with or without TAT-Gap19/DQP-1105 or NMO astrocytes with or without LA/glutamate were analyzed by real-time PCR. The protein level of AVP **(D)** and VIP **(E)** were detected by ELISA kits. *P<0.05, ***P<0.001, compared with the indicated group. N=3.

To evaluate the role of Cx43-glutamate signaling in the NMO-induced circadian rhythm dysregulation, we treated normal astrocytes with a specific Cx43 hemichannel (Cx43 HC) inhibitor TAT-Gap19 (20 μM) or a glutamate N-methyl-d-aspartate receptor (NMDAR) noncompetitive antagonist DQP-1105 (20 μM). Accordingly, the protein level of Cx43 was decreased by TAT-Gap19 mildly, whereas significantly decreased by DQP-1105. We also treated NMO-induced astrocytes with a Cx43 activator Linoleic acid (LA, 50 μM) or glutamate (50 μM) (**Figure 4A**). The results showed that the NMO decreased Cx43 protein level was reversed by LA or glutamate treatment (**Figure 4A**). The levels of glutamate in astrocytes were decreased by TAT-Gap19 and DQP-1105 treatment (**Figure 4B**). However, the suppressed glutamate levels in NMO astrocytes were elevated by LA or glutamate treatment (**Figure 4B**). Meanwhile, the levels of Clock mRNA were decreased by TAT-Gap19 and DQP-1105 treatment in normal astrocytes (**Figure 4C**) and elevated by LA or glutamate treatment in NMO astrocytes (**Figure 4C**). However, the levels of Bmal1 mRNA were unchanged with the DQP-1105 compared with blank group,and with glutamate treatment compared with NMO group (**Figure 4C**). The levels of VIP protein were decreased by DQP-1105 treatment in the co-culture medium of normal astrocytes and neurons (**Figure 4D**) and elevated by LA or glutamate treatment in the co-culture medium of NMO astrocytes (**Figure 4D**). However, the levels of AVP protein were unchanged with these treatments (**Figure 4E**). Collectively, these results demonstrated that Cx43-glutamate signaling impairment is critical to NMO-induced circadian rhythm dysregulation.

### 3.4 Bmal1 knockdown reversed the Cx43-glutamate signaling activation

**Figure 5:**
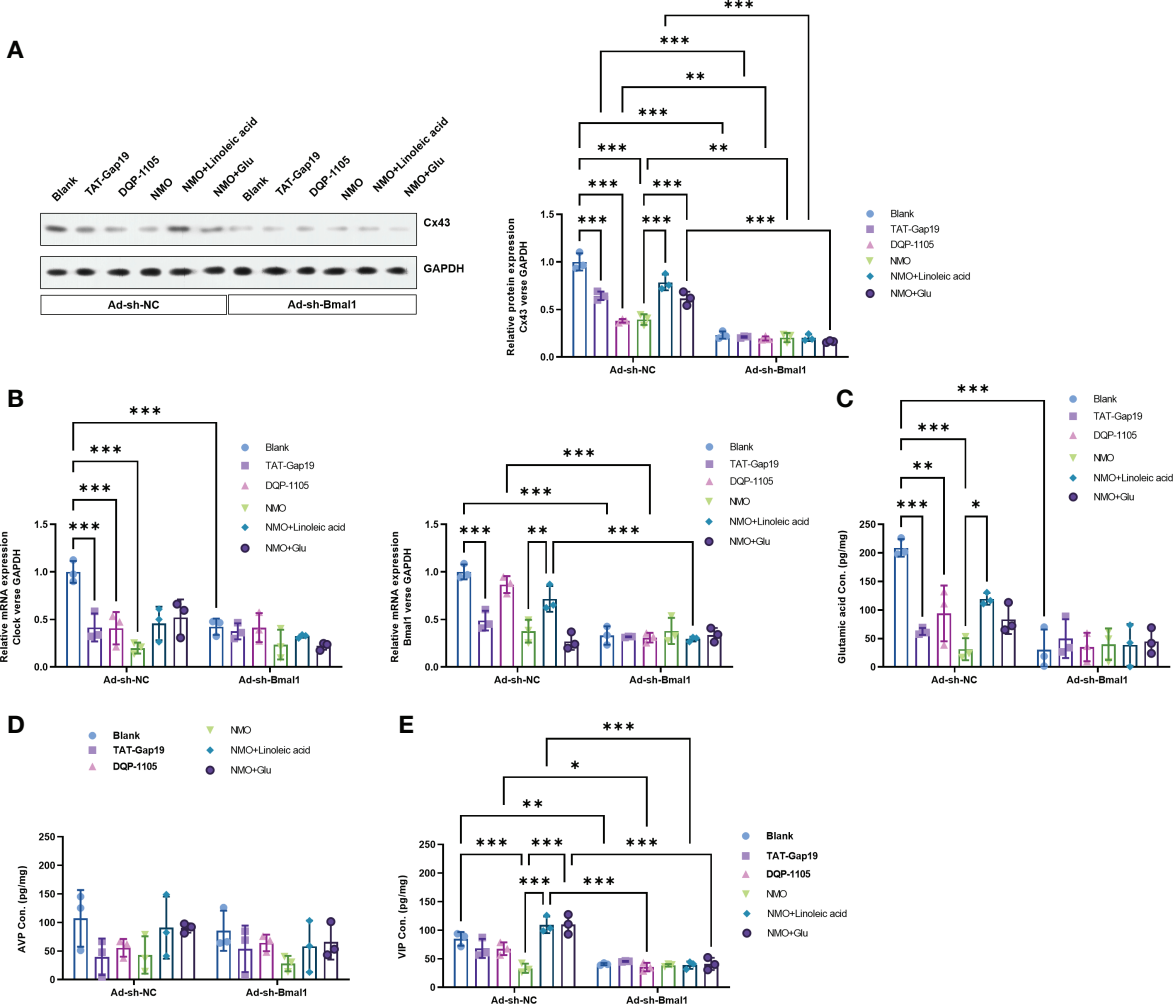
Bmal1 knockdown reversed the Cx43-glutamate signaling activation. **(A)** The effect of Bmal1 knock-down on the protein level of Cx43 in normal astrocytes with or without TAT-Gap19/DQP-1105 or NMO astrocytes with or without LA/glutamate were analyzed by Western Blot. **(B)** The effect of Bmal1 knock-down on the mRNA levels of Clock and Bmal1 in normal astrocytes with or without TAT-Gap19/DQP-1105 or NMO astrocytes with or without LA/glutamate were analyzed by real-time PCR. **(C)** The effect of Bmal1 knock-down on the glutamate levels in normal astrocytes with or without TAT-Gap19/DQP-1105 or NMO astrocytes with or without LA/glutamate were analyzed by ELISA kit. The effect of Bmal1 knockdown on the protein level of AVP **(D)** and VIP **(E)** was detected by ELISA kits. *P<0.05, **P<0.01, ***P<0.001, compared with the indicated group. N=3.

To explore the critical role of Bmal1 in the Cx43-glutamate signaling impairment in NMO-induced circadian rhythm dysregulation, we constructed a recombinant adenovirus containing a short hairpin RNA targeting Bmal1 to knock down the expression of Baml1 in astrocytes. Interestingly, Bmal1 knock-down significantly inhibited the protein level of Cx43 in the normal astrocytes or NMO astrocytes, which was neither changed by the treatment of TAT-Gap19 and DQP-1105 in normal astrocytes nor the treatment of LA or glutamate treatment in NMO astrocytes (**Figure 5A**). Moreover, the mRNA expression level of Bmal1 and Clock (**Figure 5B**) and the glutamate levels (**Figure 5C**) were also suppressed by Bmal1 knockdown, which was neither changed by the treatment of TAT-Gap19 and DQP-1105 in normal astrocytes nor the treatment of LA or glutamate treatment in NMO astrocytes. Correspondingly, the levels of AVP protein from neurons were also suppressed by Bmal1 knockdown, which was neither changed by the co-culture of TAT-Gap19 and DQP-1105 treated normal astrocytes nor the co-culture of LA or glutamate treated NMO astrocytes (**Figure 5D**). The levels of VIP protein were also unchanged with these treatments (**Figure 5E**). These results revealed that Bmal1 knockdown reversed the Cx43-glutamate signaling activation.

## 4 Discussion

The primary astrocytic disease NMO is associated with inflammation and secondary myelin loss in the CNS (1, 2). Aquaporin 4 (AQP4), a water channel, plays an important role in disease pathogenesis as a target of autoantibodies (NMO-IgG) in patient sera (19). Astrocytes are the major source of AQP4 expression in the brain, especially near the end feet, where the blood-brain barrier is located (4). As a result of the interaction between NMO-IgG and AQP4 in astrocytes, rapid AQP4 endocytosis initiates pathogenesis (7) and induced cortical neurodegeneration (41). In this study, we constructed an anti-AQP4 IgG-induced NMO model of SCN astrocytes. We found that the Cx43-glutamate signaling and circadian rhythm regulating genes (Bmal1 and Clock) were significantly decreased in a time-dependent manner.

Connexin proteins are involved in the formation of homotypic or heterotypic gap junctions (GJs) between astrocytes, or between astrocytes and oligodendrocytes. GJs connect two cells and provide direct intercellular communication. They are responsible for the exchange of intracellular second messengers, such as calcium ions. Cx43 levels declined in half of the NMO cases, almost parallel with the diminution of AQP4 levels in active lesions (42). Astroglial Cx43 protein plays a significant role in several CNS functions, including cognitive behavior, motor control, and sleep-wake regulation. Circadian rhythms control sleep and wakefulness (43). It is the SCN that generates the central circadian rhythm, and it is closely coupled with the rest of the brain to generate coherence (44). During the circadian cycle, the SCN is entrained in the environmental light-dark cycle through the excitation of glutamatergic neurons (38). A key role of connexins is to regulate the rhythmic activity of neuronal activity in SCN by electric coupling of neurons and astrocytic-neuronal signaling (37). However, the role of Cx43 in the regulation of circadian rhythms regulating genes, including Bmal1 and Clock, remains largely unclear in SCN. Herein, we found that the levels of Bmal1 and Clock were significantly decreased in normal astrocytes with a specific Cx43 hemichannel (Cx43 HC) inhibitor TAT-Gap19. Furthermore, the level of Clock was increased in the NMO-induced astrocytes with a Cx43 activator LA.

Cx43 is the most abundant connexin expressed on astrocytes and forms gaps between astrocytes, which are the most abundant and most functional glial cells in the brain (45). Because Cx43 hemichannels can pass through large molecules, their opening might provide a mechanism for transmitters like glutamate to diffuse out of astrocytes (46, 47). As a result of its adaptive advantage, the circadian clock allows for predictive, rather than entirely reactive, homeostatic regulation of physiological functions. It was reported that the Circadian clock-regulated ATP release from Cx43 might contribute in part to the adaptation of functional bladder capacity in daily life. Moreover, it was reported that astrocytic Cx43 was down-regulated in human NMO lesions (48) and an *in vitro* model (49). In NMO, a large number of inflammatory infiltrates could be seen in NMO lesions (50, 51). On the other hand, inflammatory cytokines could also suppress astrocytic Cx43 (52). In this study, we further found that the levels of Bmal1 and Clock were significantly decreased in normal astrocytes with a glutamate N-methyl-d-aspartate receptor (NMDAR) noncompetitive antagonist DQP-1105. The level of Clock was increased in the NMO-induced astrocytes with glutamate. These changes supposed that the circadian rhythms may be regulated by Cx43-signaling in the pathogenesis of NMO.

It is well known that sleep and circadian rhythm are closely intertwined, and they are coordinated to adapt the organism to varying environments (29). The sleep abnormalities associated with NMOSD patients have been mentioned in previous reports (31). Based on some observation results regarding clinical phenomena and the specific regions of damage of NMOSD, it appears that rhythmic damage occurs more easily (17, 32). However, the underlying mechanism of rhythmic damage and NMO were rarely reported. From our observations, on the one hand, the NMO induction dramatically decreased the rhythm-regulating genes, including Bmal1 and Clock, which were increased by Cx43 activator LA and glutamate, suggesting that Cx43-glutamate signaling would be a critical upstream regulator that contributes to the NMO-induced rhythmic damage in SCN astrocytes. On the other hand, our results suggested that the decreasing of Bmal1, the critical gene in circadian rhythms, contributes to the disease aggressiveness and circadian rhythm disorders of NMO.

The limitation of this study is that the *in vitro* environment cannot completely simulate the complex *in vivo* environment. Therefore, *in vivo* experiments need to be perfected to further verify the results.

In conclusion, our experiment confirmed that clock gene Bmal1 and Cx43 mediate inflammatory regulation and circadian rhythm bidirectionally in NMO. Cx43 of astrocytes is involved in NMO circadian rhythm disorders by influencing the diurnal oscillation of glutamate. Activation of Cx43-glutamate signaling would be a promised strategy for the therapy of NMOSD and prevent NMO-damaged circadian rhythms.

## Data availability statement

The original contributions presented in the study are included in the article/**Supplementary Material**. Further inquiries can be directed to the corresponding authors.

## Ethics statement

The animal study was reviewed and approved by Ethics Committee of the First Hospital of Shanxi Medical University.

## Author contributions

MZ and HZ put forward research ideas and sets overall research goals. HX organizes and analyzes the data and is responsible for writing the article. MW, YW, and YZ performs the specific operation of the experiment. All authors contributed to the article and approved the submitted version.
