# Supplementary figures and images for: The circadian rhythms regulated by Cx43-signaling in the pathogenesis of Neuromyelitis Optica
Source: Front Immunol. 2023 Jan 16;13:1021703. doi: 10.3389/fimmu.2022.1021703 (PMC9885795; doi:10.3389/fimmu.2022.1021703)

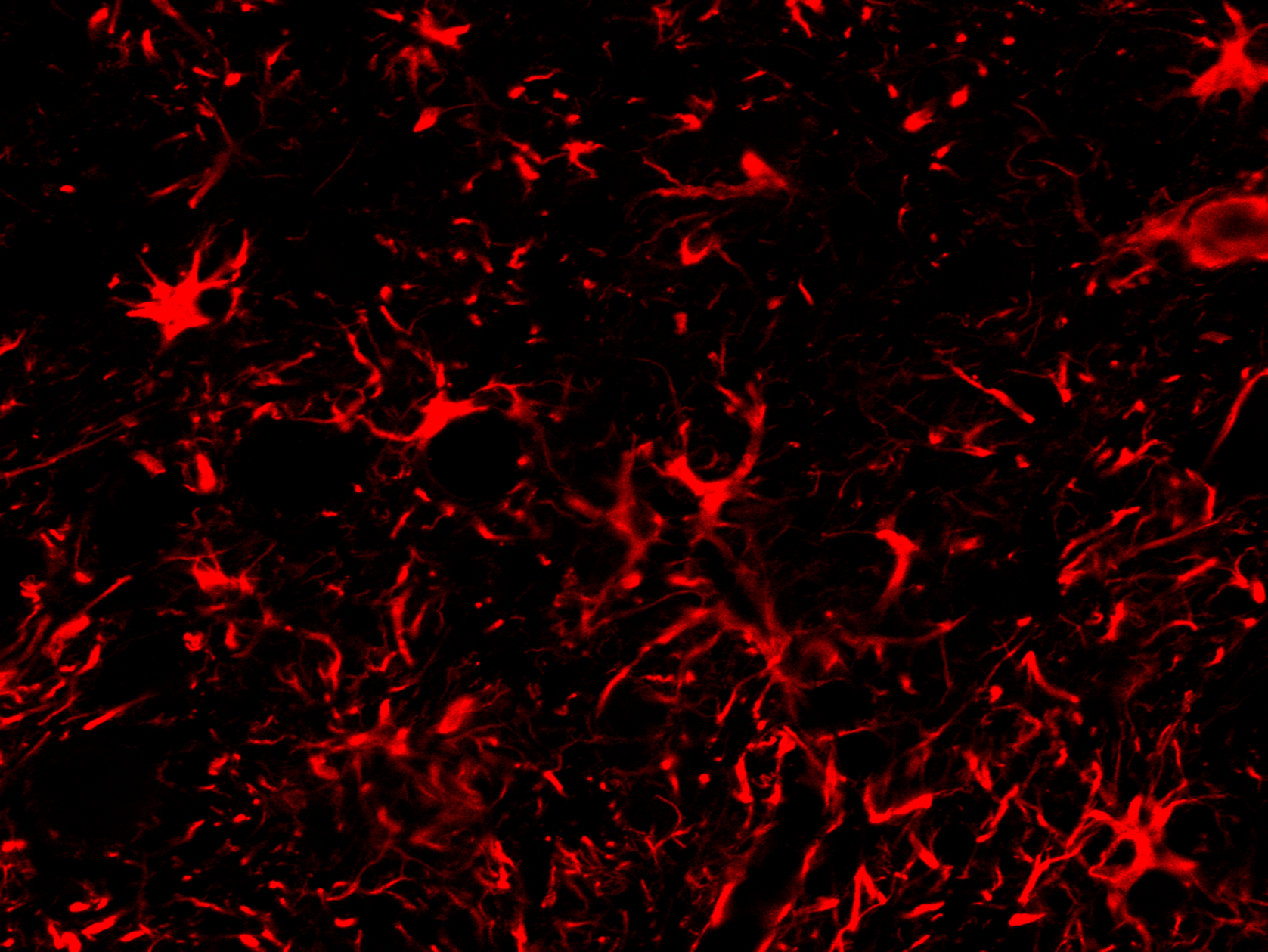

Supplement: Supplementary file 1 [file DataSheet_1.zip › Part 1/ASTs-GFAP-IF/Part 1-ASTs-GFAP-IF (1).tif]

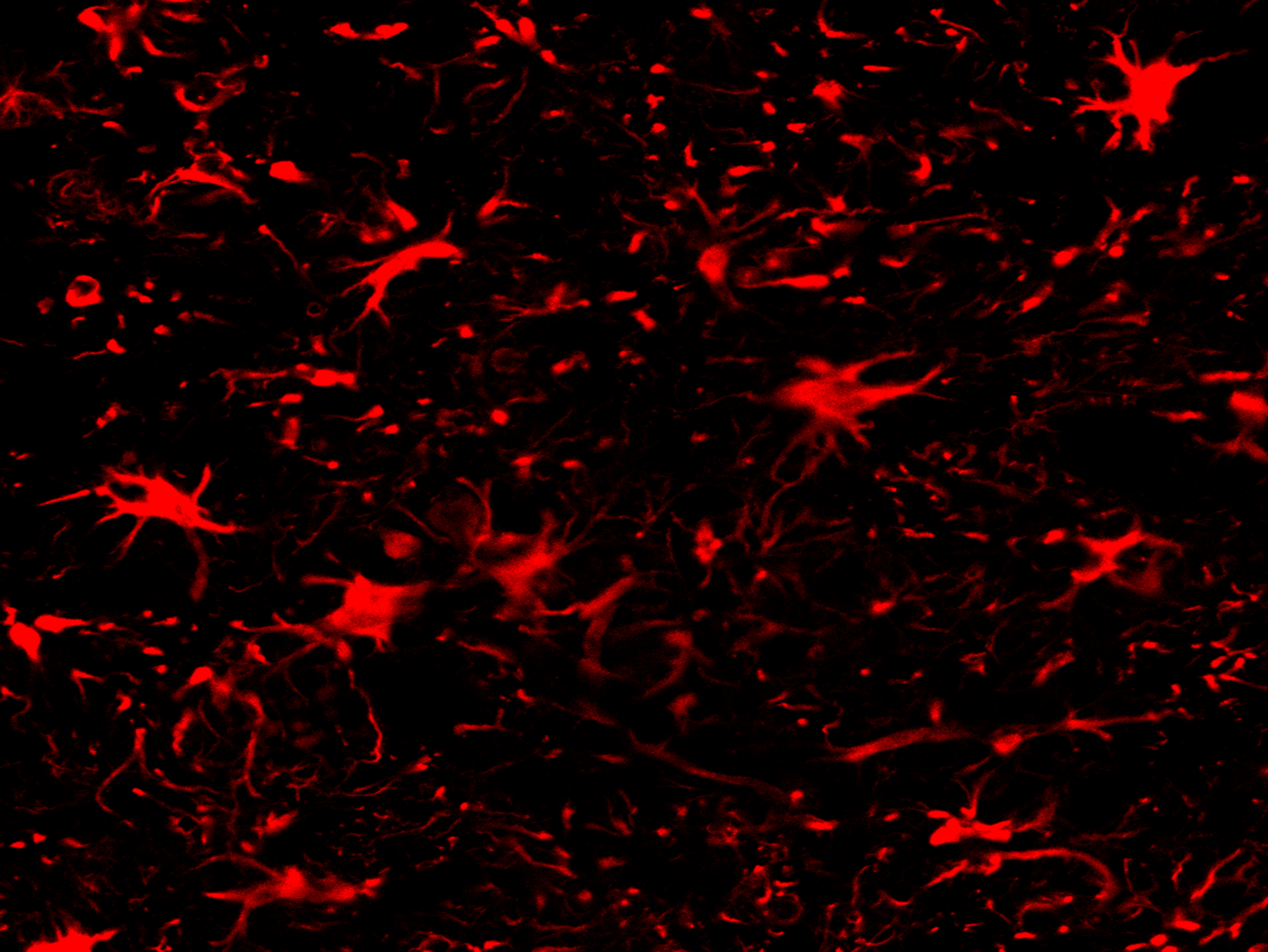

Supplement: Supplementary file 1 [file DataSheet_1.zip › Part 1/ASTs-GFAP-IF/Part 1-ASTs-GFAP-IF (2).tif]

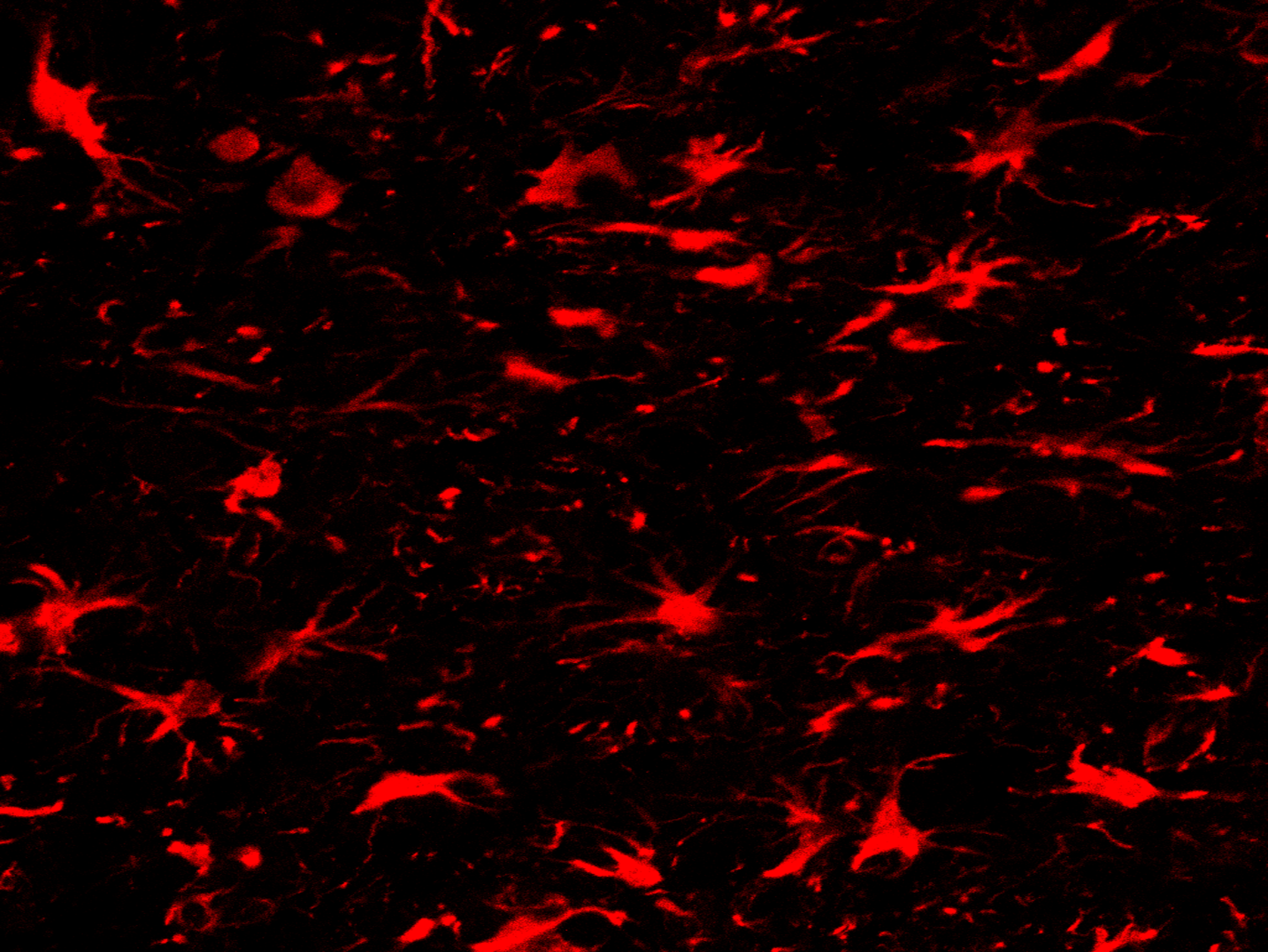

Supplement: Supplementary file 1 [file DataSheet_1.zip › Part 1/ASTs-GFAP-IF/Part 1-ASTs-GFAP-IF (3).tif]

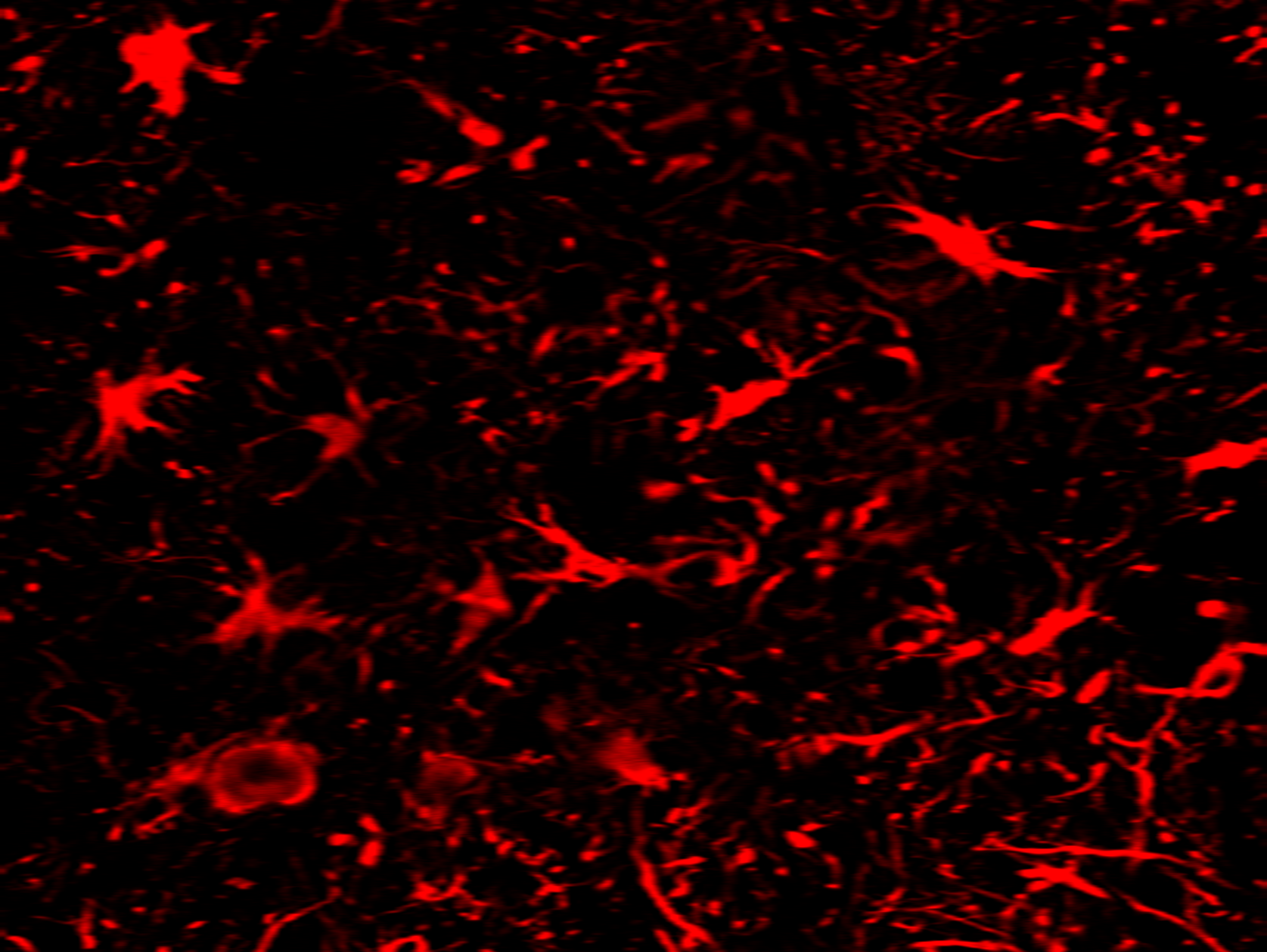

Supplement: Supplementary file 1 [file DataSheet_1.zip › Part 1/ASTs-GFAP-IF/Part 1-ASTs-GFAP-IF (4).tif]

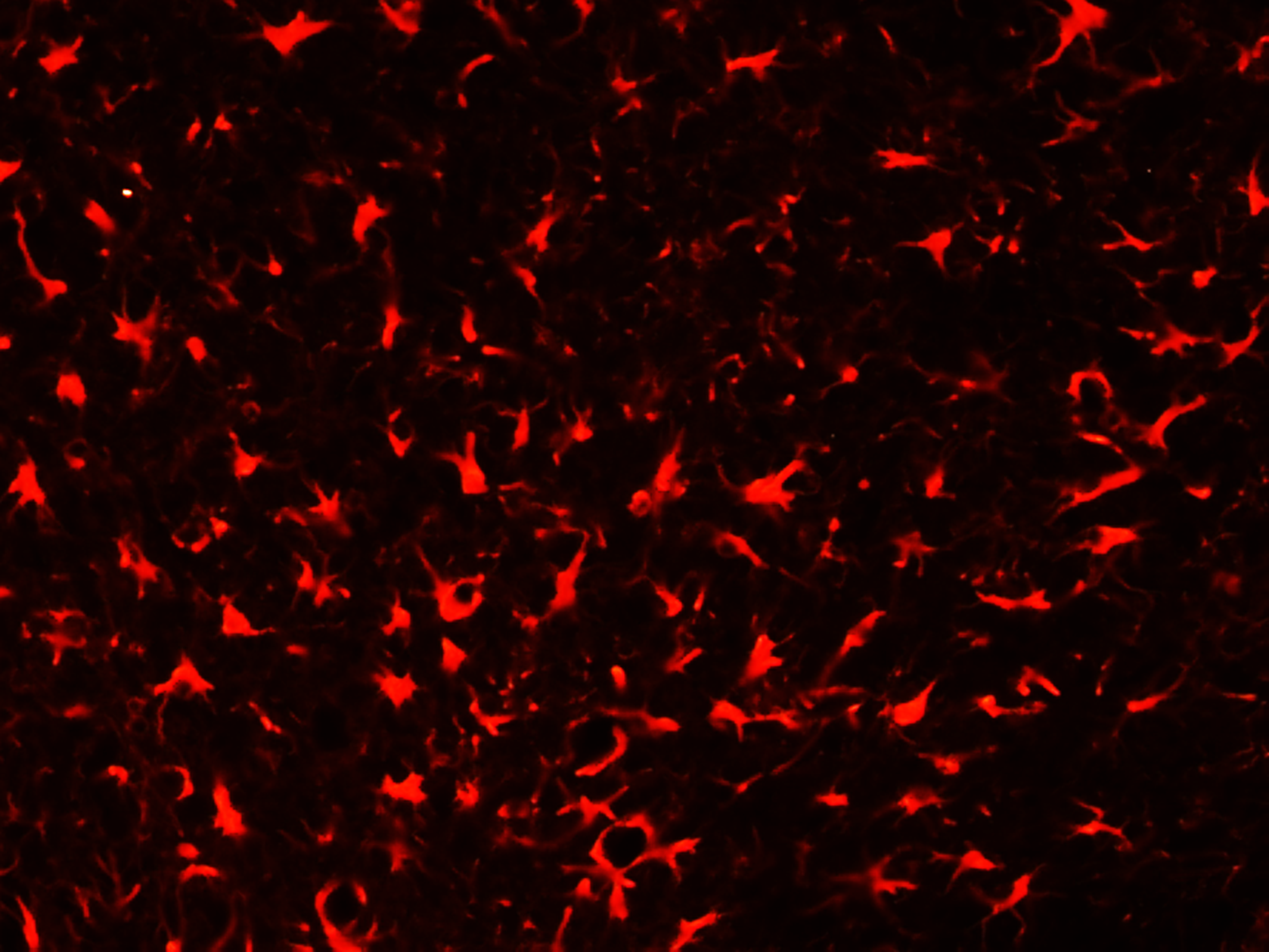

Supplement: Supplementary file 1 [file DataSheet_1.zip › Part 1/ASTs-GFAP-IF/Part 1-ASTs-GFAP-IF (5).tif]

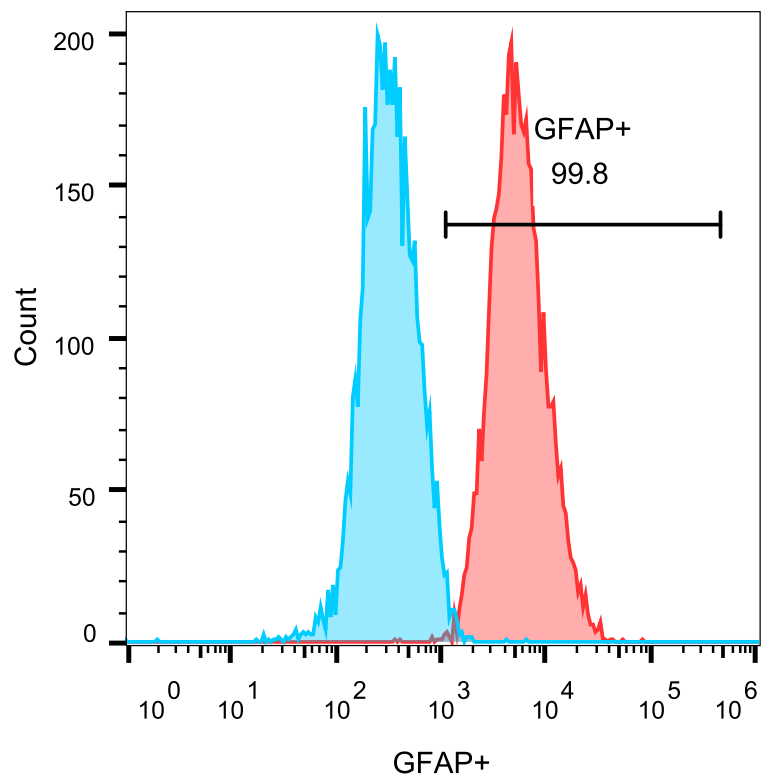

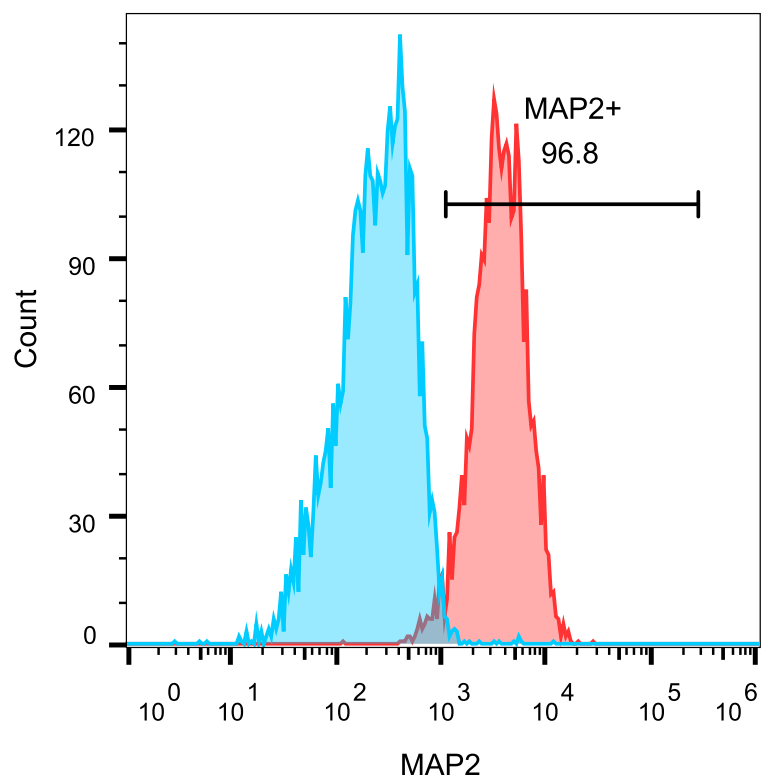

Supplement: Supplementary file 1 [file DataSheet_1.zip › Part 1/FACs/Part 1-Positive rate of detection by Flow cytometry.pdf]

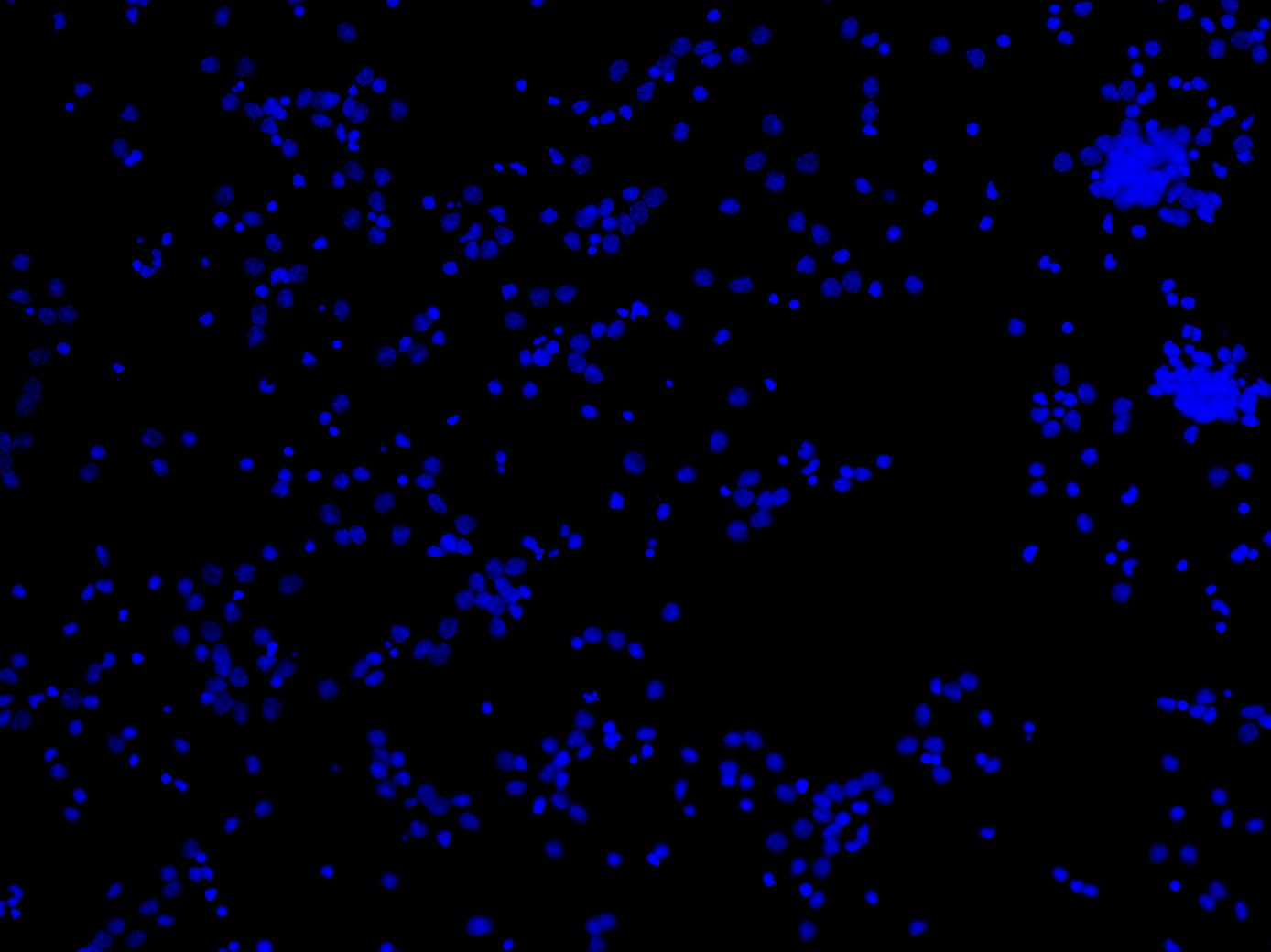

Supplement: Supplementary file 1 [file DataSheet_1.zip › Part 1/Neuron MAP2 IF/Part 1-Neuron-DAPI (5).tif]

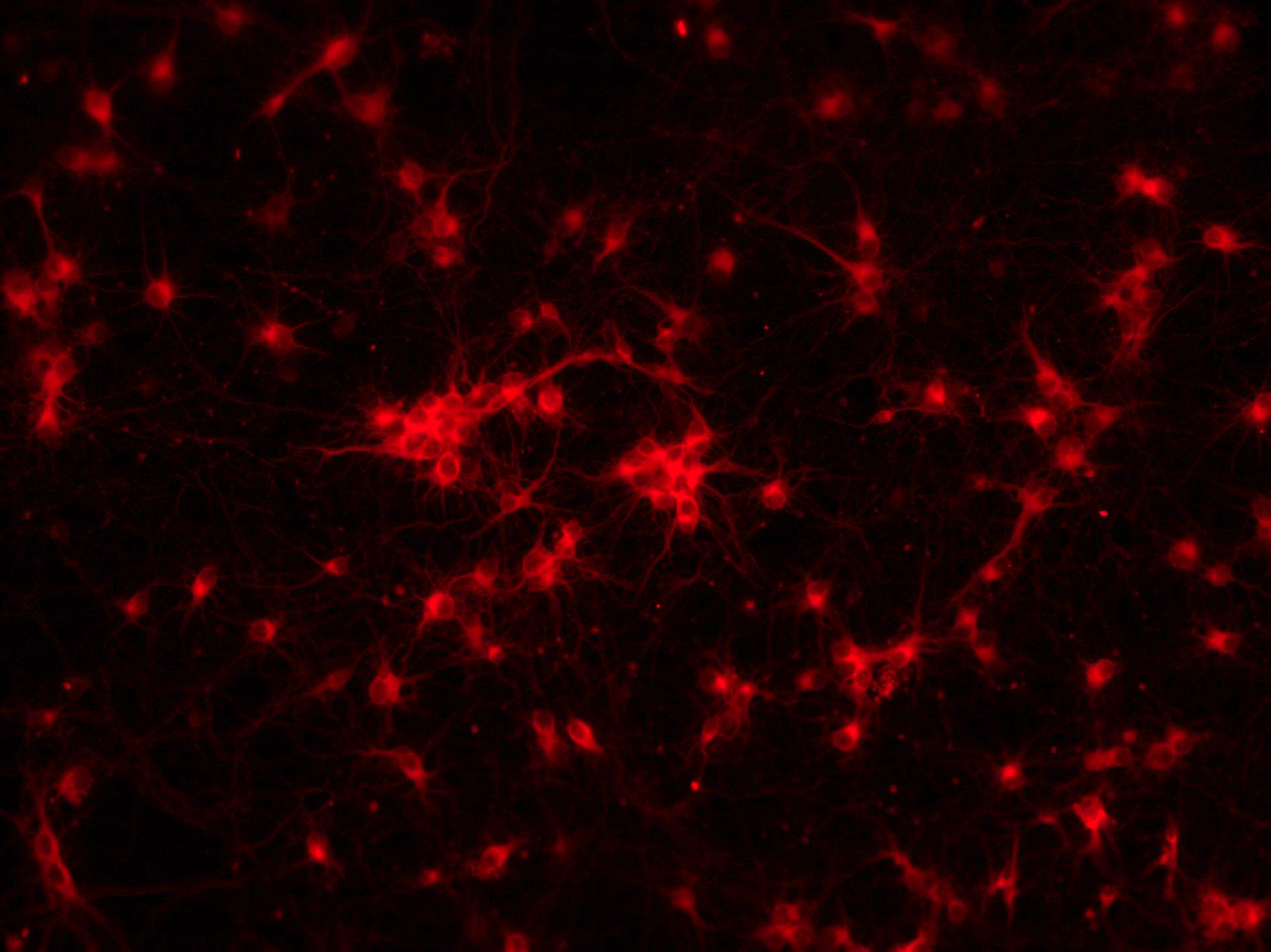

Supplement: Supplementary file 1 [file DataSheet_1.zip › Part 1/Neuron MAP2 IF/Part 1-Neuron-MAP2 (1).tif]

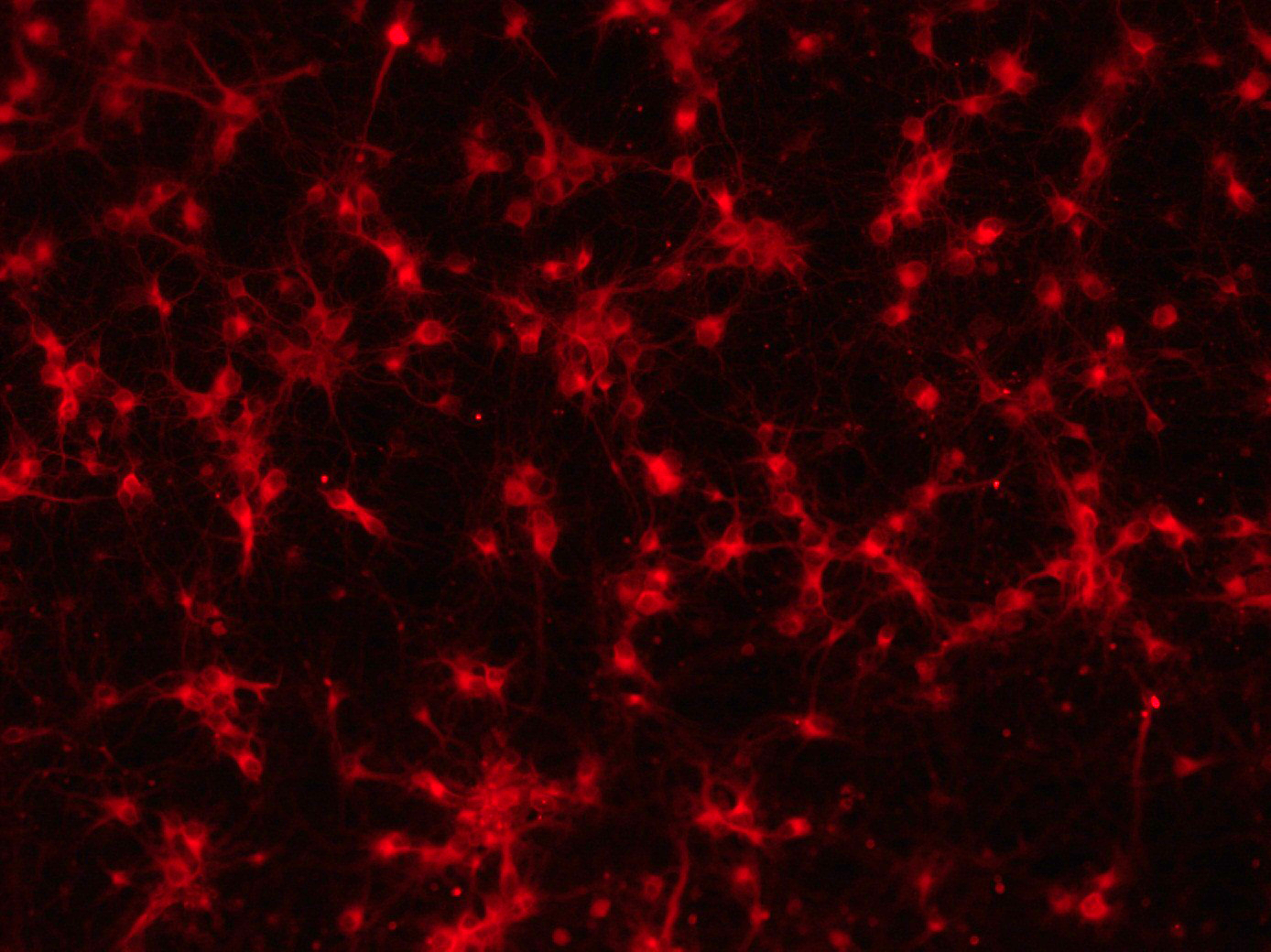

Supplement: Supplementary file 1 [file DataSheet_1.zip › Part 1/Neuron MAP2 IF/Part 1-Neuron-MAP2 (2).tif]

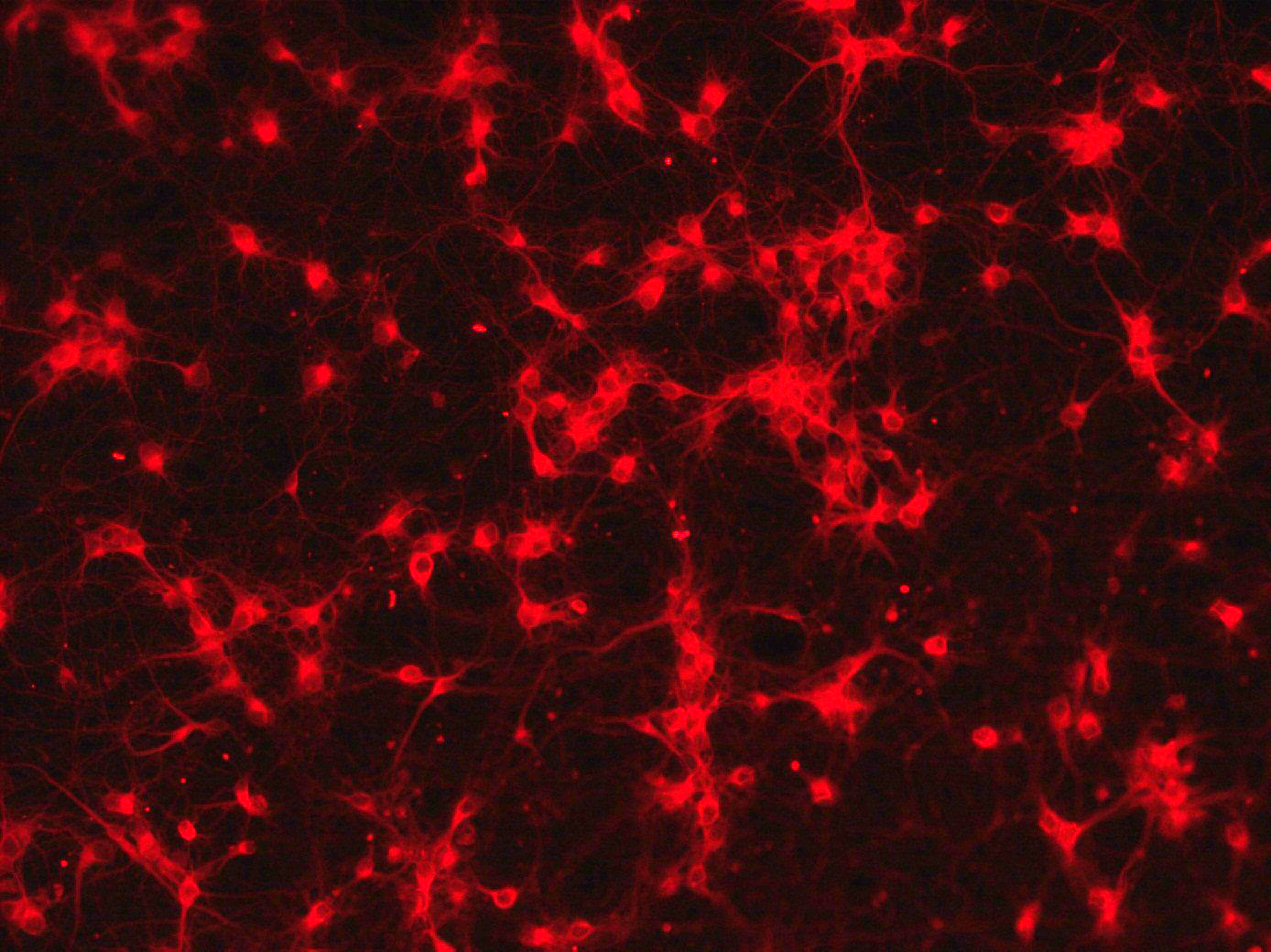

Supplement: Supplementary file 1 [file DataSheet_1.zip › Part 1/Neuron MAP2 IF/Part 1-Neuron-MAP2 (3).tif]

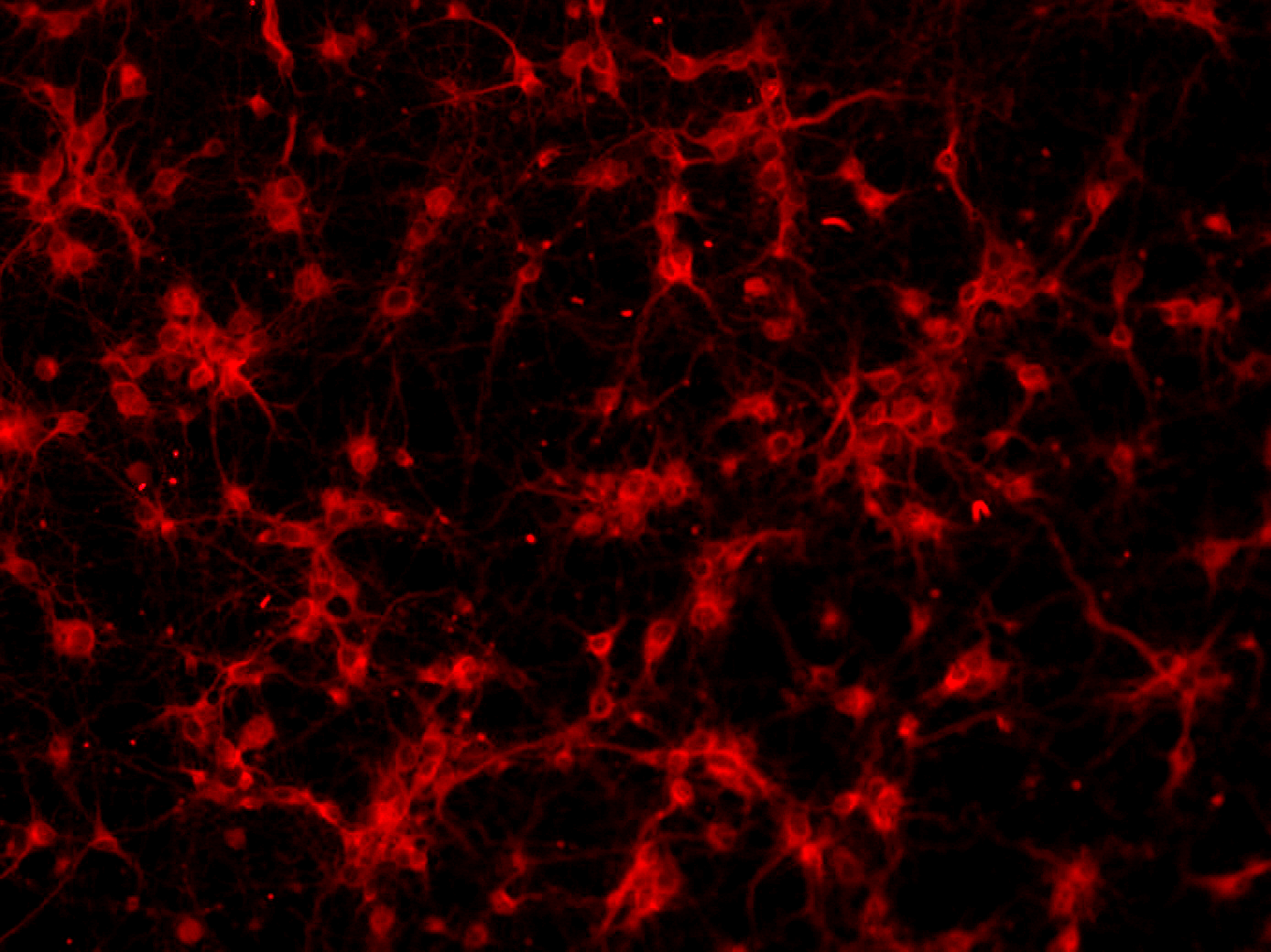

Supplement: Supplementary file 1 [file DataSheet_1.zip › Part 1/Neuron MAP2 IF/Part 1-Neuron-MAP2 (4).tif]

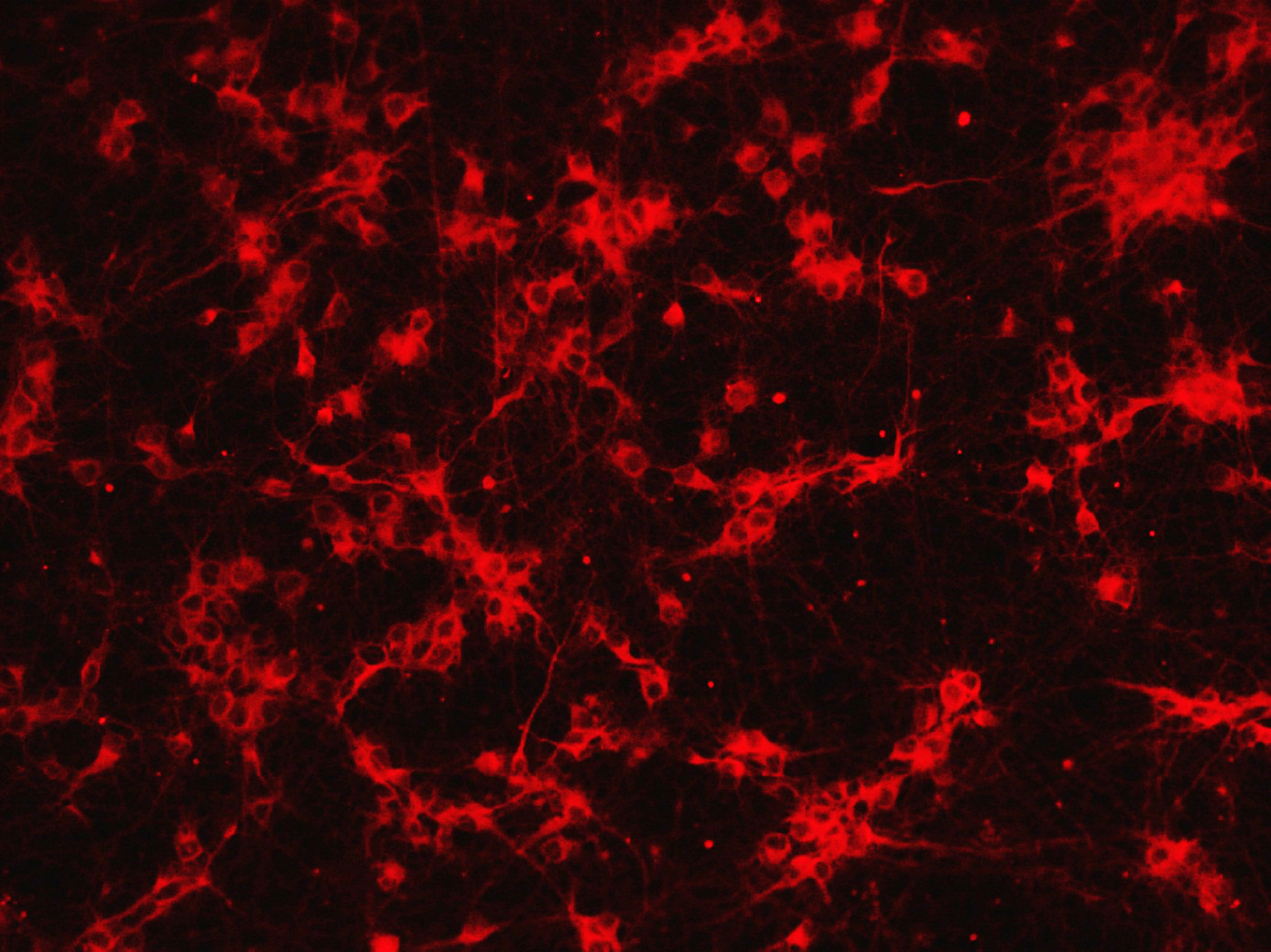

Supplement: Supplementary file 1 [file DataSheet_1.zip › Part 1/Neuron MAP2 IF/Part 1-Neuron-MAP2 (5).tif]

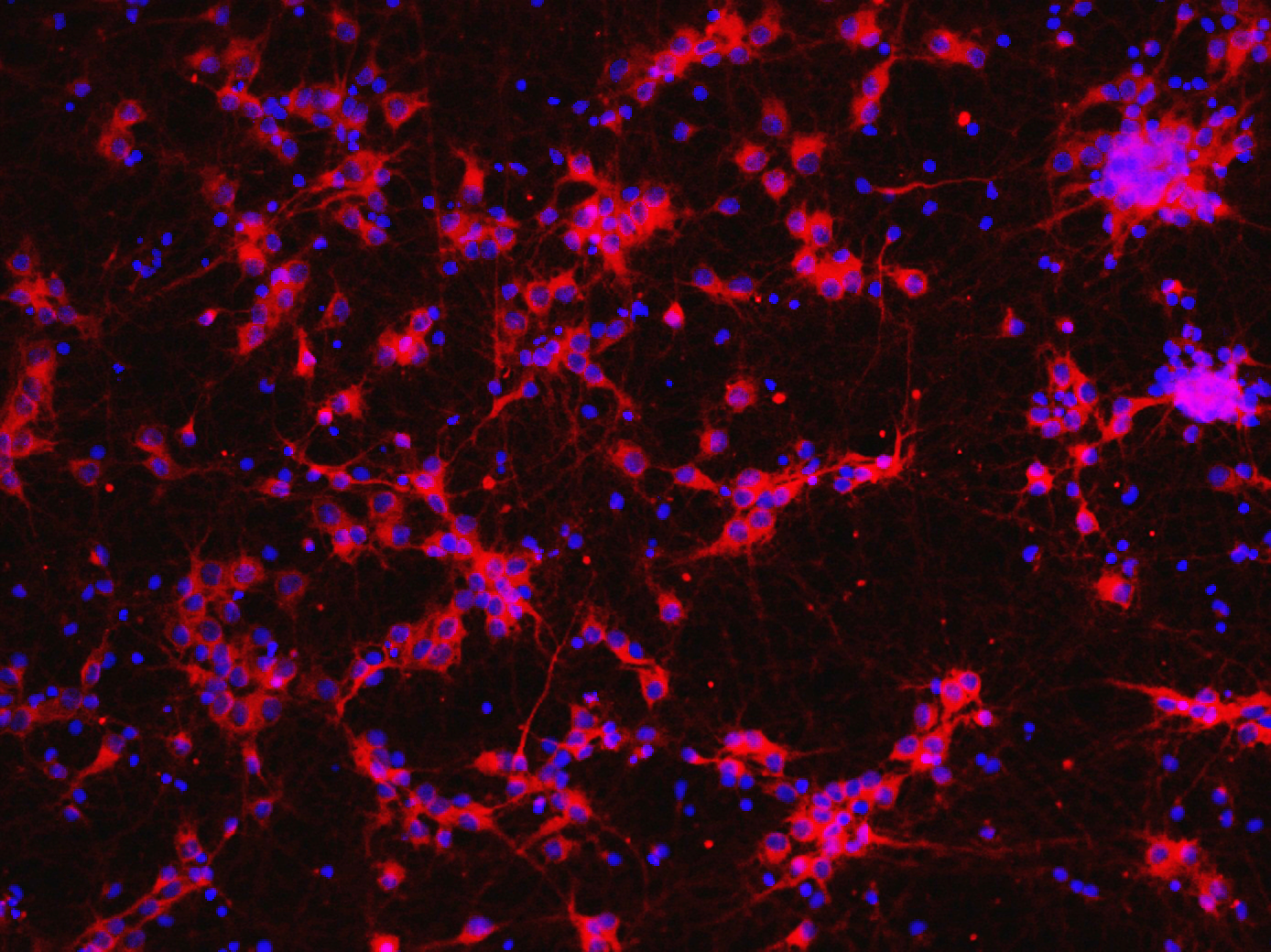

Supplement: Supplementary file 1 [file DataSheet_1.zip › Part 1/Neuron MAP2 IF/Part 1-Neuron-Merge (5).tif]

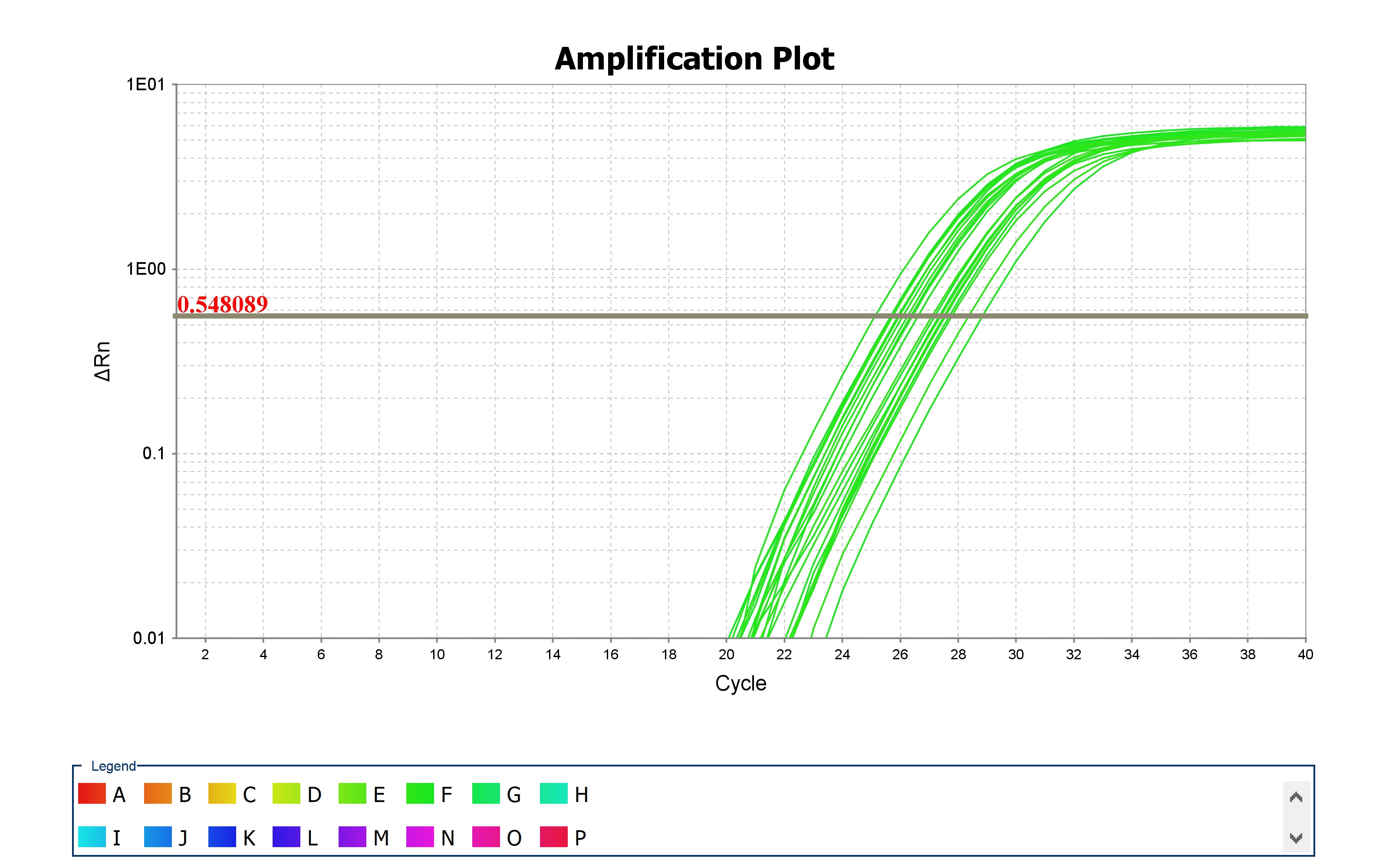

Supplement: Supplementary file 1 [file DataSheet_1.zip › Part 1/Real-time PCR/Amplification Plot-Baml1.jpg]

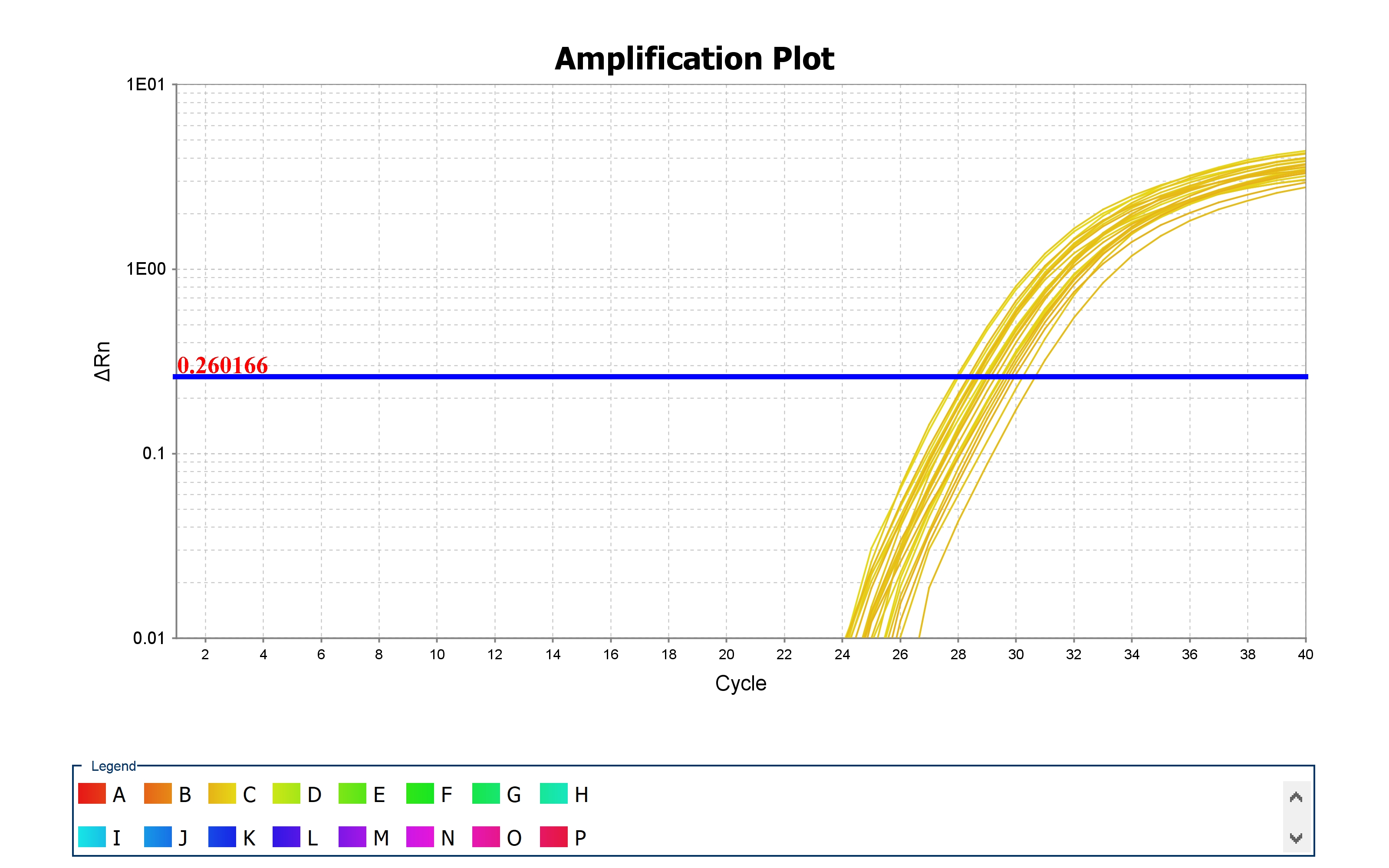

Supplement: Supplementary file 1 [file DataSheet_1.zip › Part 1/Real-time PCR/Amplification Plot-CLOCK.jpg]

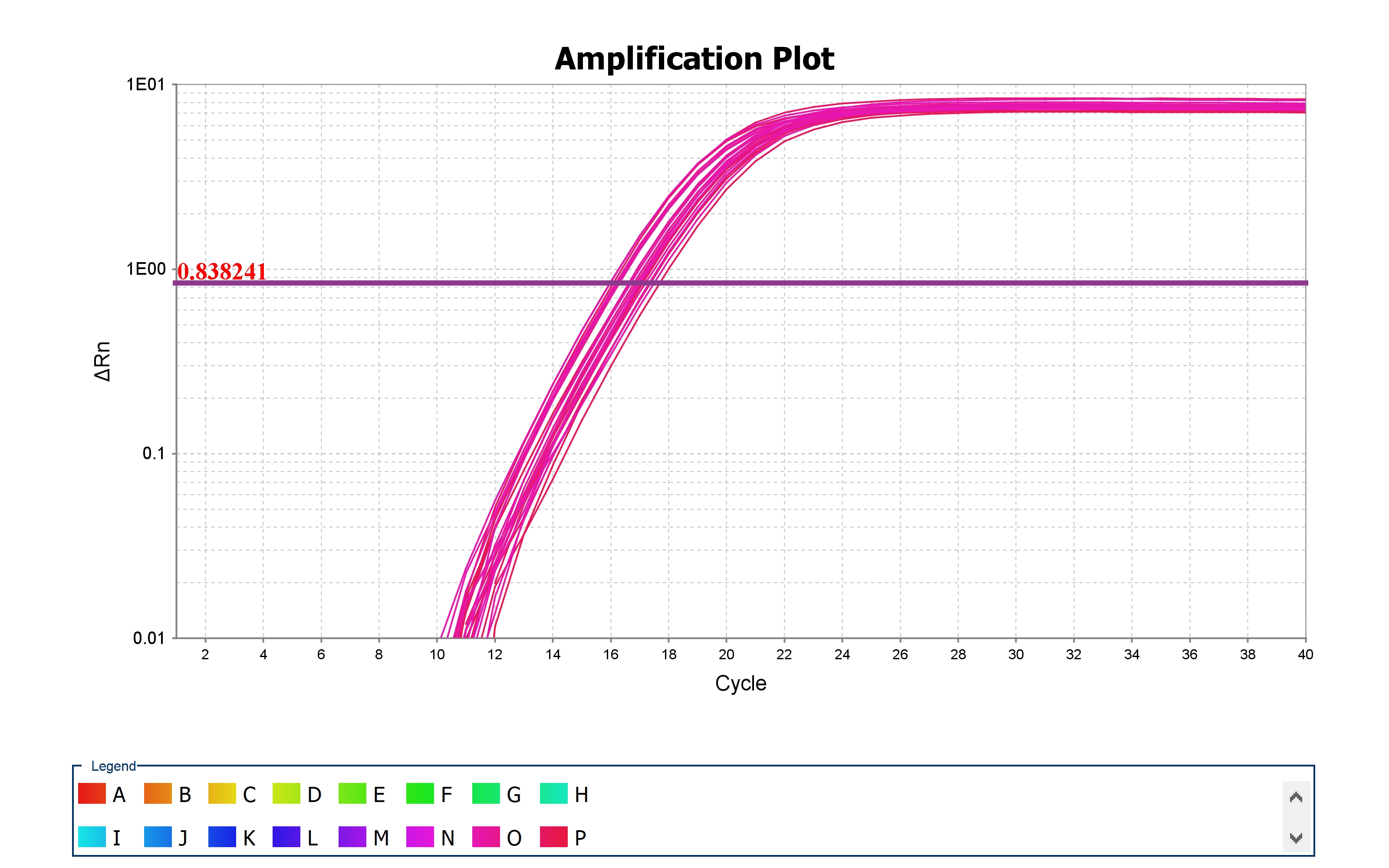

Supplement: Supplementary file 1 [file DataSheet_1.zip › Part 1/Real-time PCR/Amplification Plot-GAPDH.jpg]

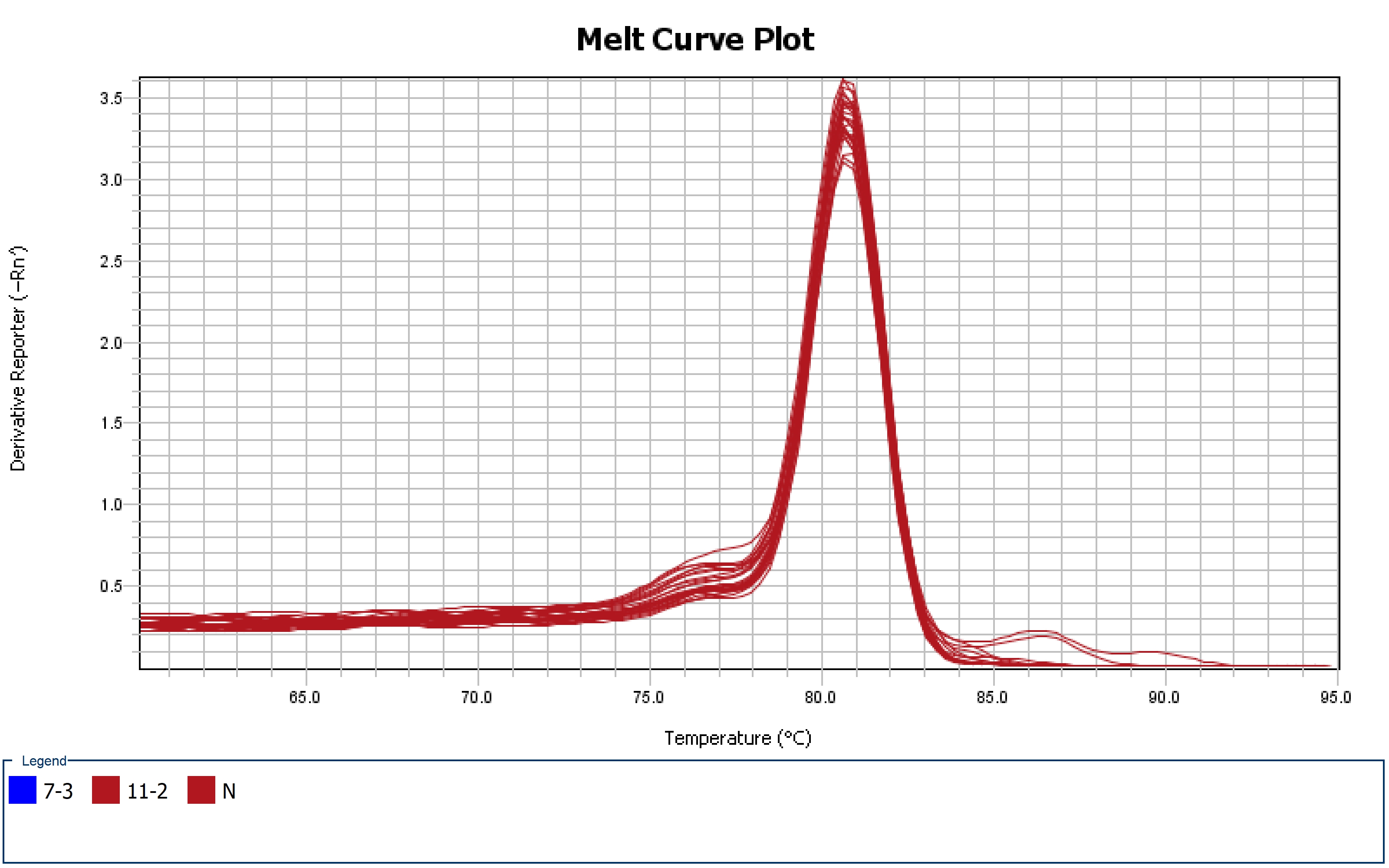

Supplement: Supplementary file 1 [file DataSheet_1.zip › Part 1/Real-time PCR/Melt Curve Plot-Bmal1.jpg]

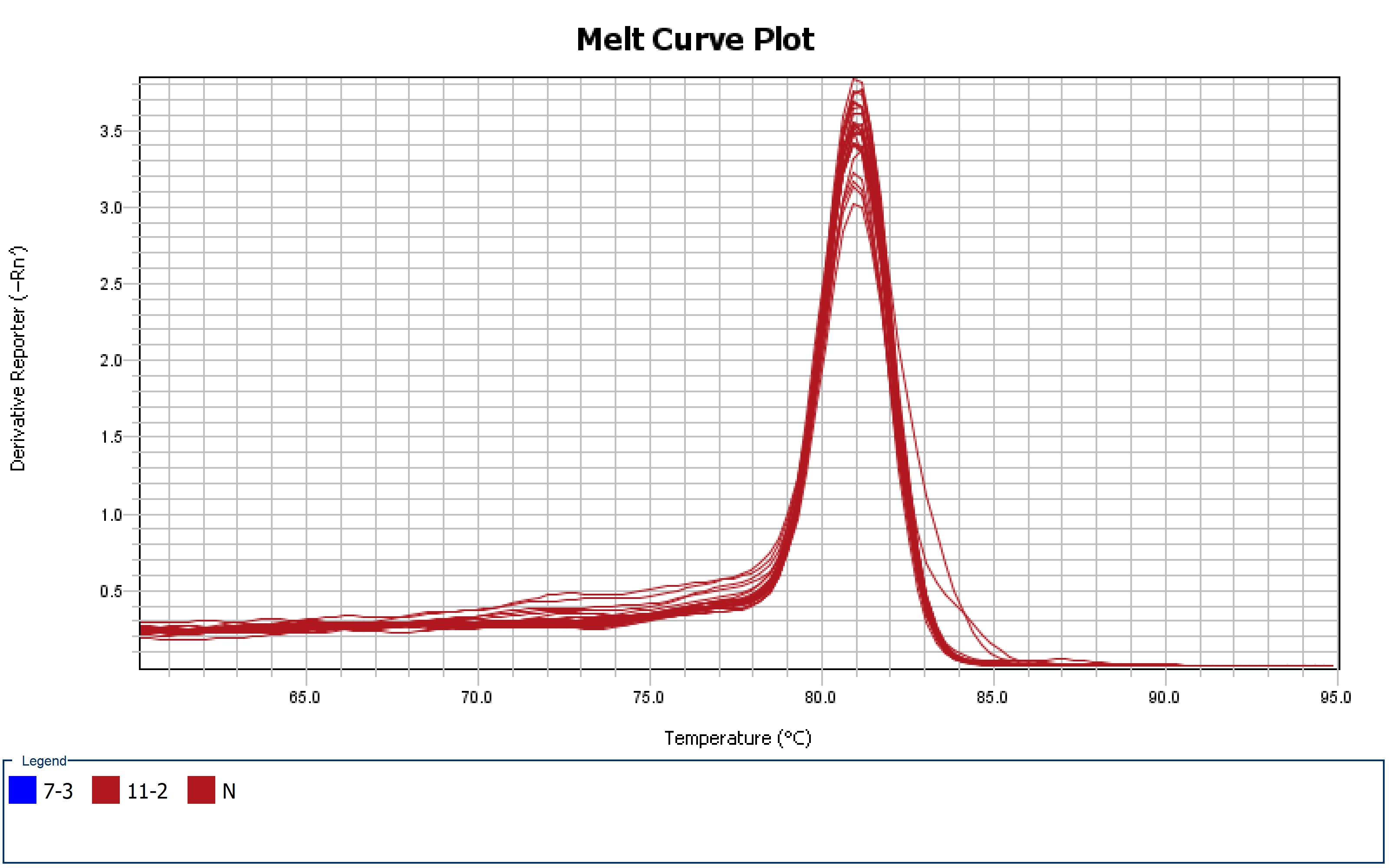

Supplement: Supplementary file 1 [file DataSheet_1.zip › Part 1/Real-time PCR/Melt Curve Plot-CLOCK.jpg]

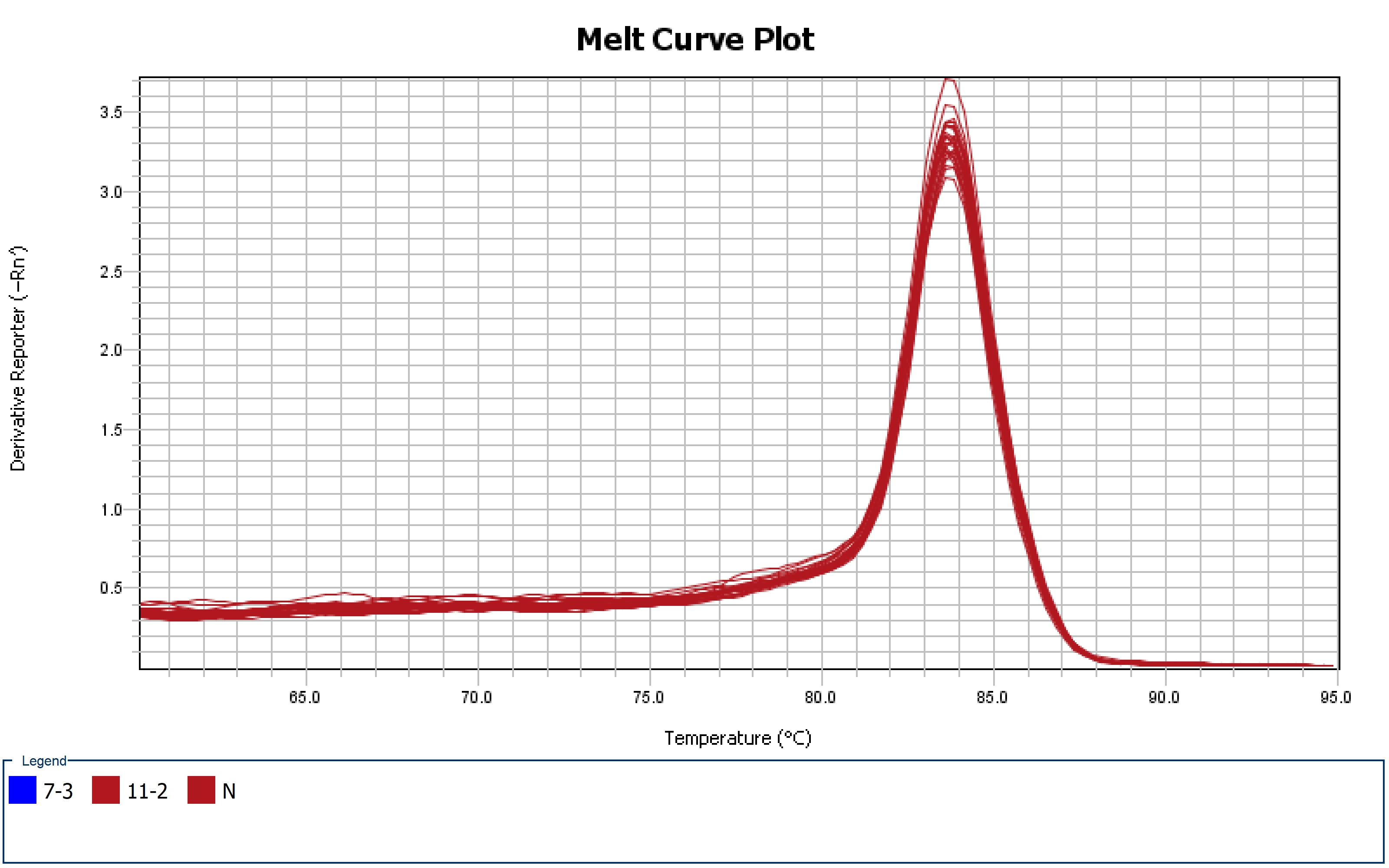

Supplement: Supplementary file 1 [file DataSheet_1.zip › Part 1/Real-time PCR/Melt Curve Plot-GAPDH.jpg]

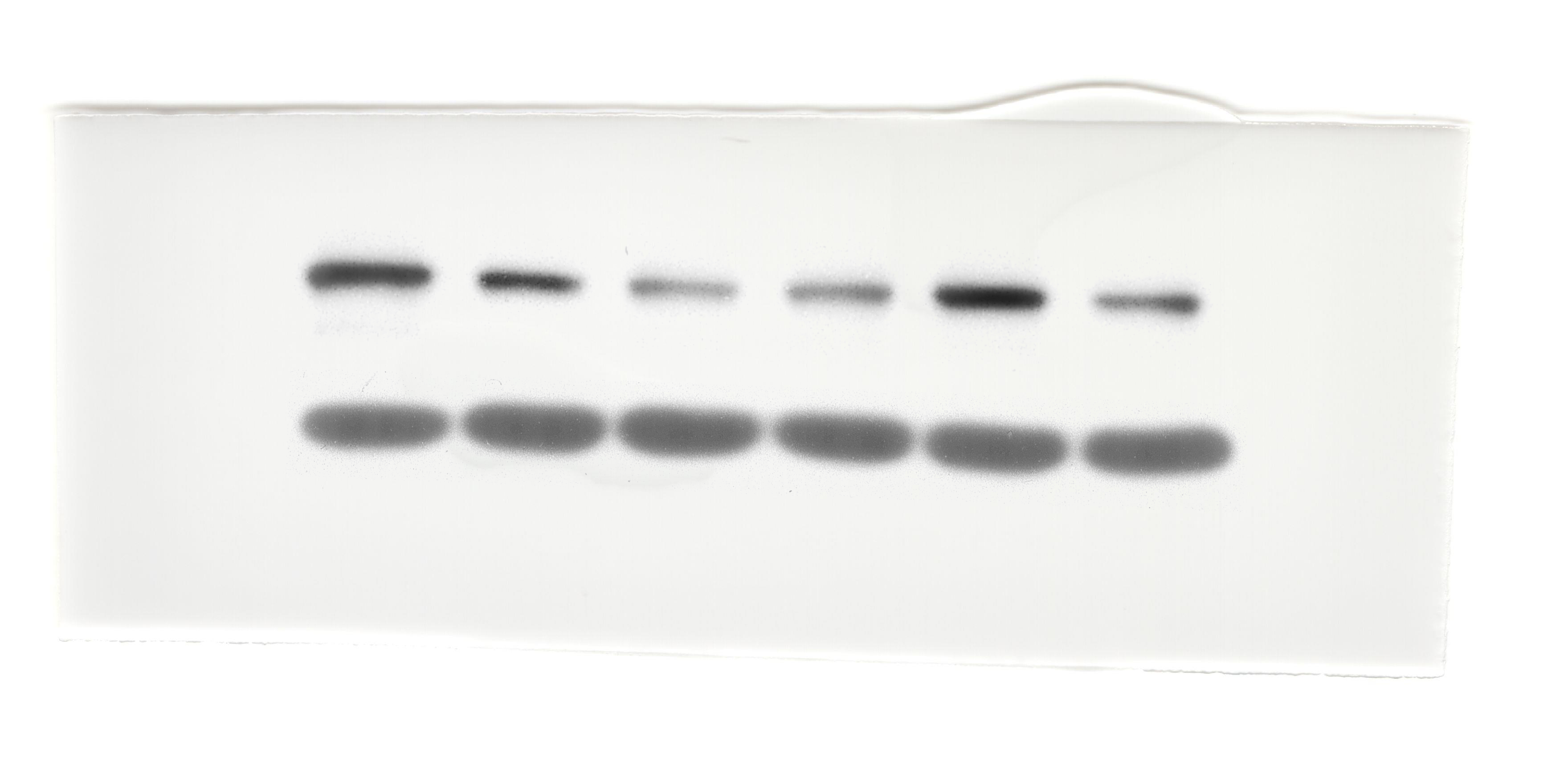

Supplement: Supplementary file 1 [file DataSheet_1.zip › Part 1/Western Blot/Part 1-CX43+GAPDH.tif]

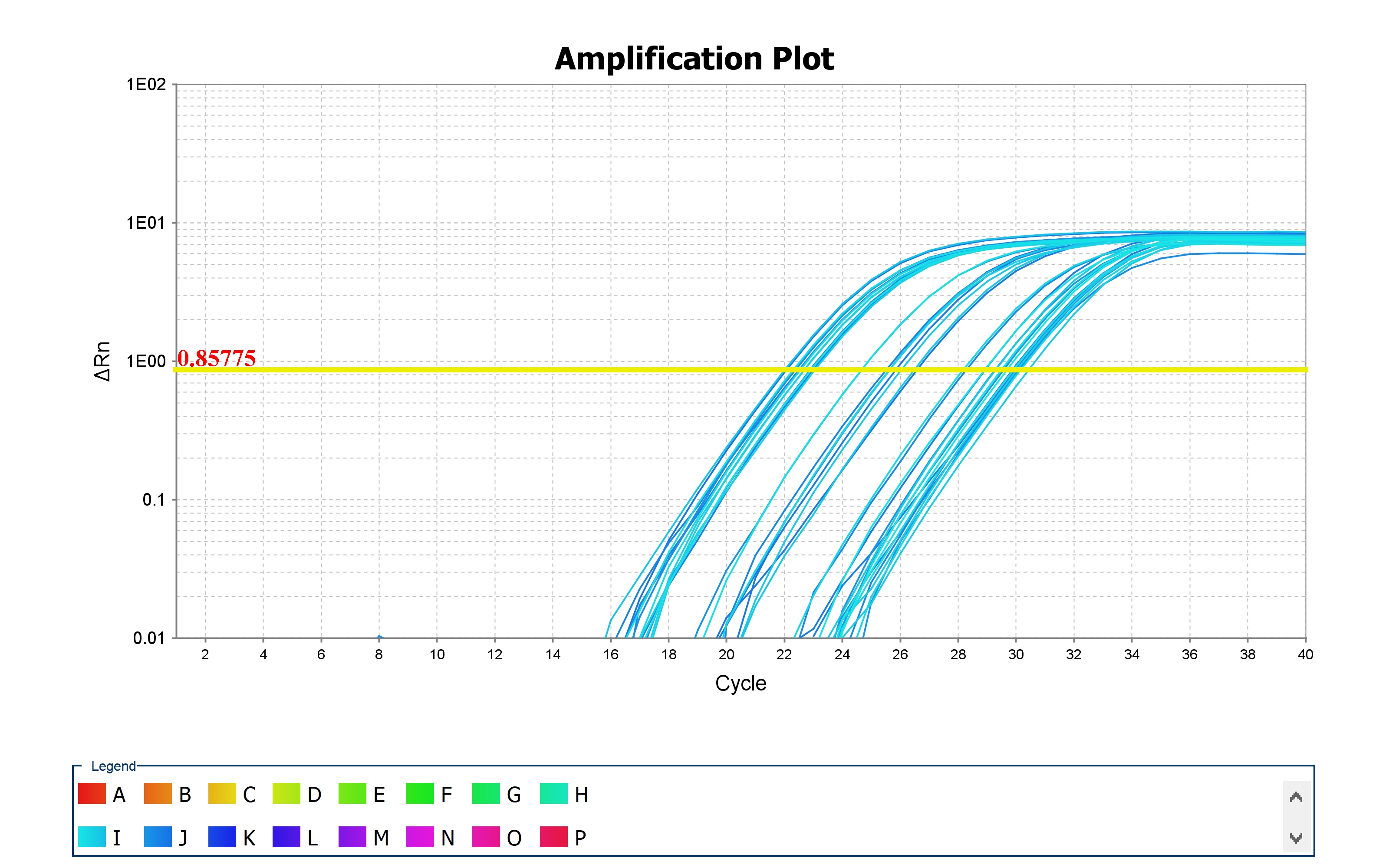

Supplement: Supplementary file 2 [file DataSheet_2.zip › Part 2/Real-time PCR/Amplification Plot-Bmal1.jpg]

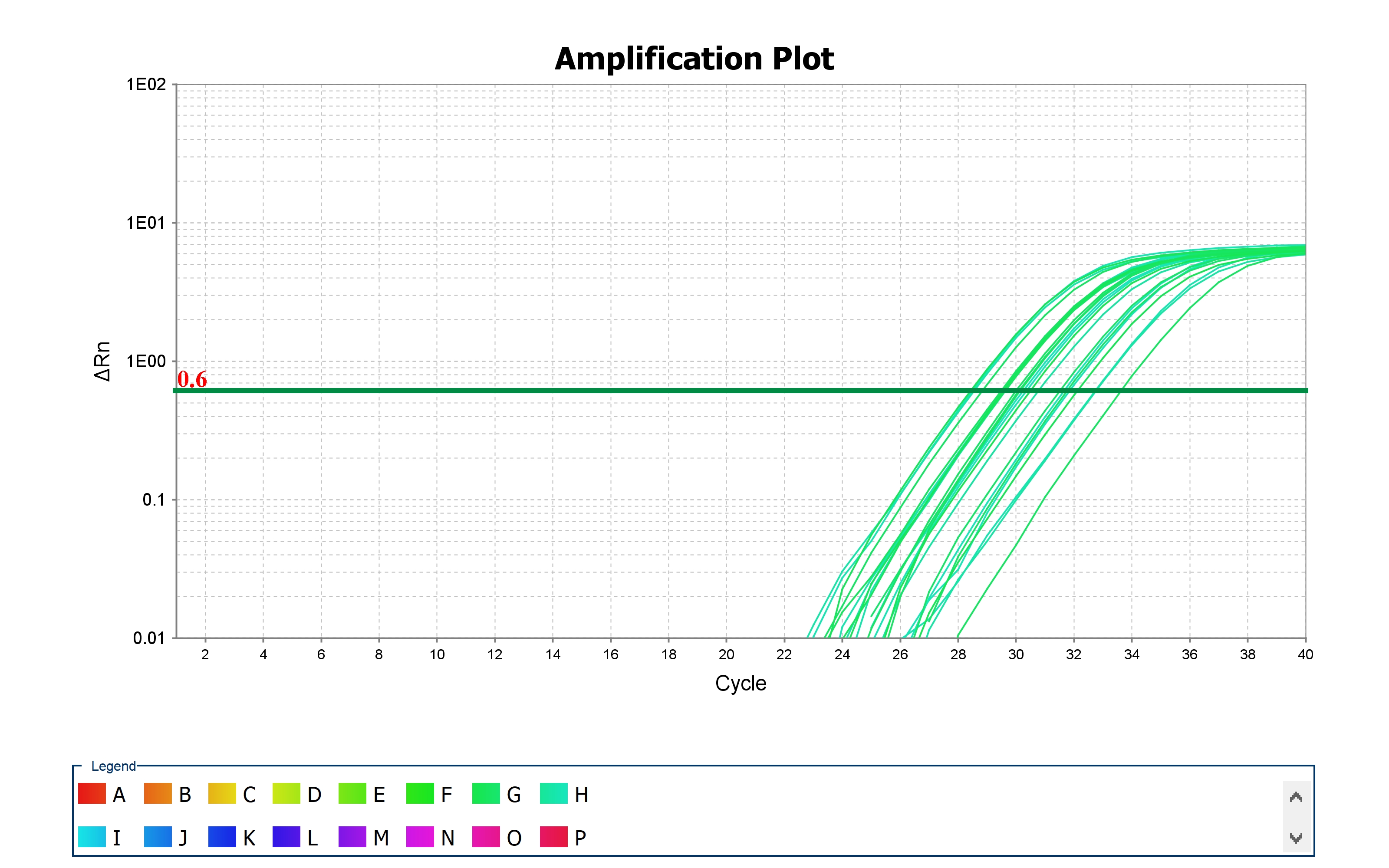

Supplement: Supplementary file 2 [file DataSheet_2.zip › Part 2/Real-time PCR/Amplification Plot-Clock.jpg]

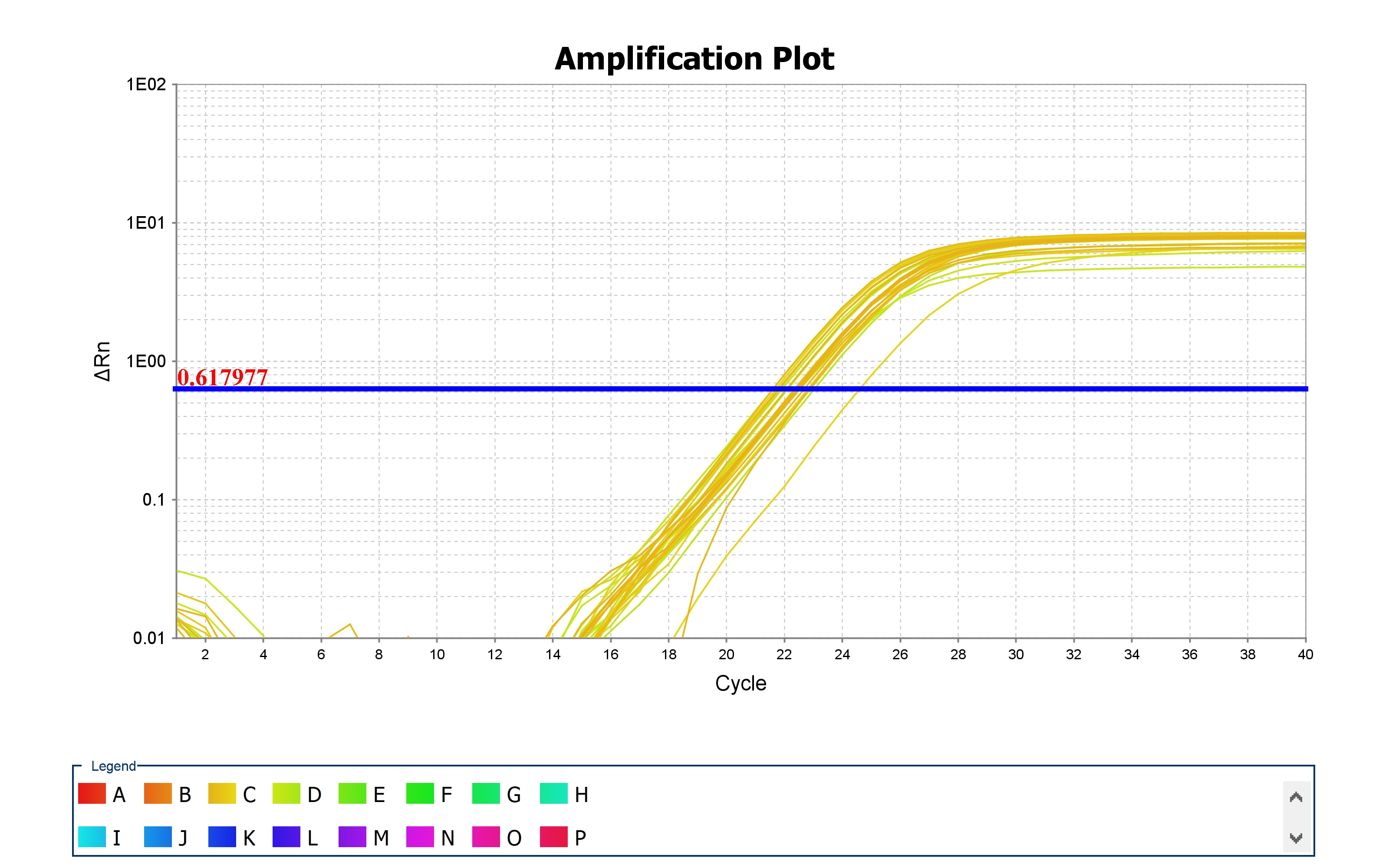

Supplement: Supplementary file 2 [file DataSheet_2.zip › Part 2/Real-time PCR/Amplification Plot-GAPDH.jpg]

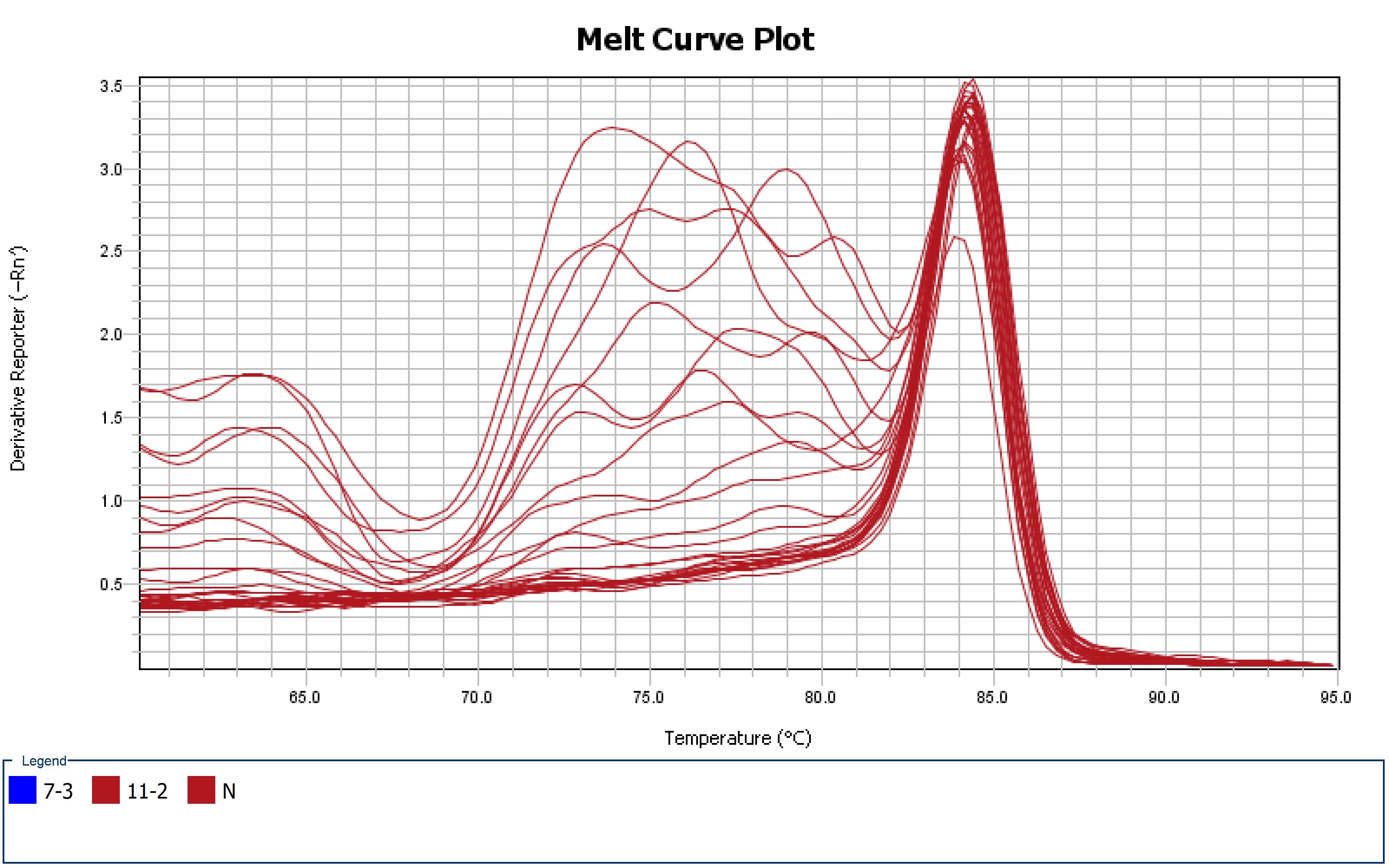

Supplement: Supplementary file 2 [file DataSheet_2.zip › Part 2/Real-time PCR/Melt Curve Plot-Bmal1.jpg]

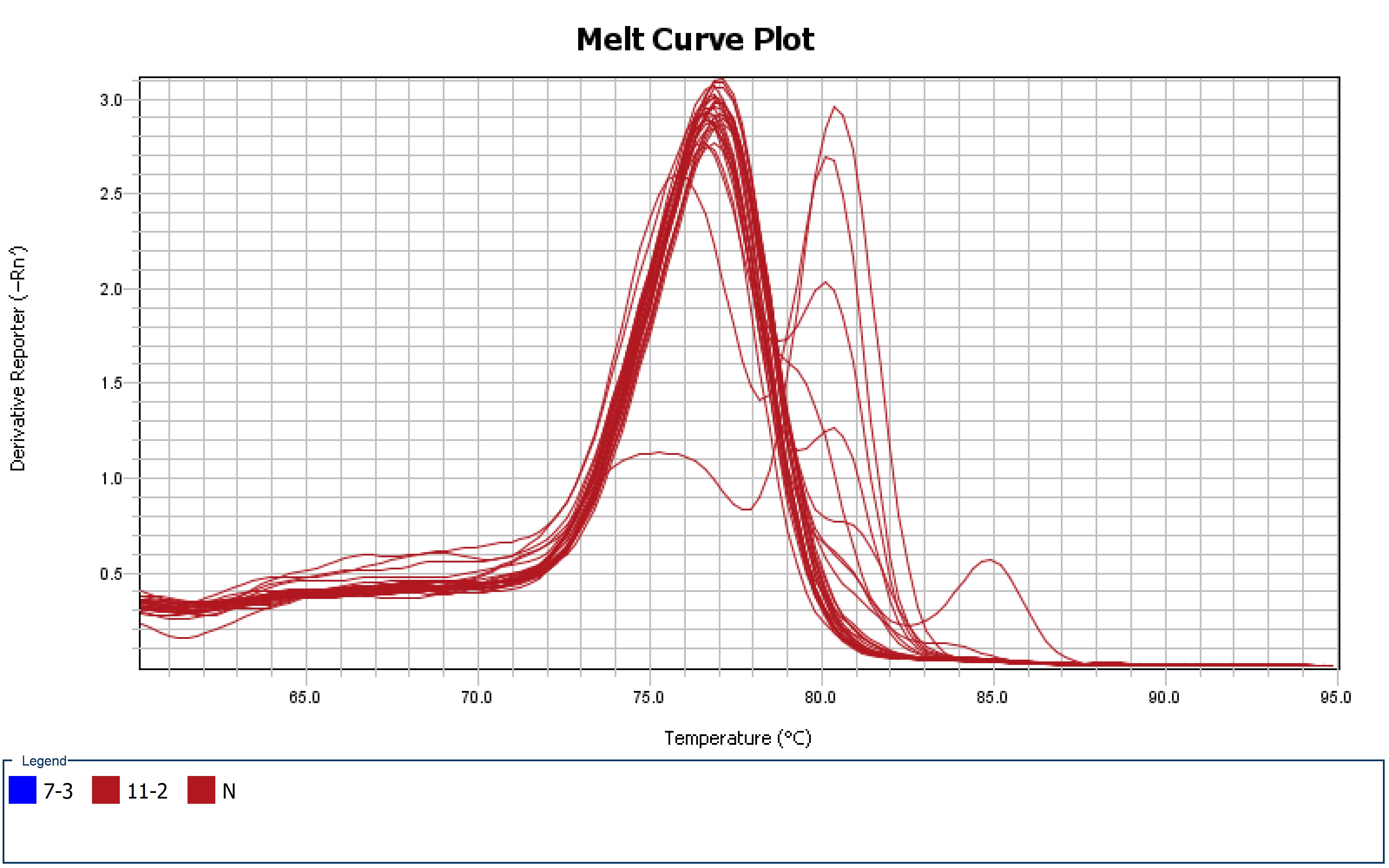

Supplement: Supplementary file 2 [file DataSheet_2.zip › Part 2/Real-time PCR/Melt Curve Plot-Clock.jpg]

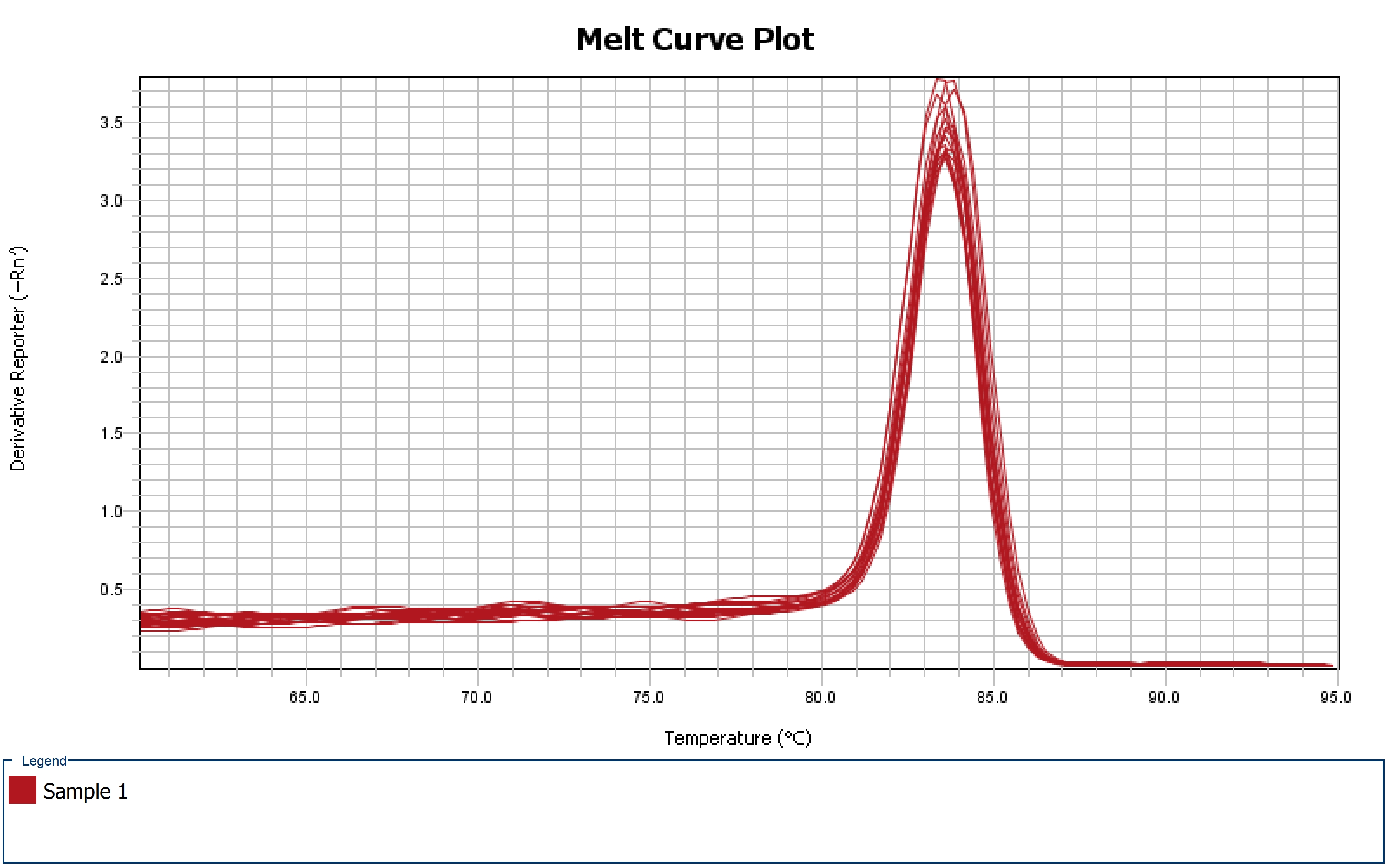

Supplement: Supplementary file 2 [file DataSheet_2.zip › Part 2/Real-time PCR/Melt Curve Plot-GAPDH.jpg]

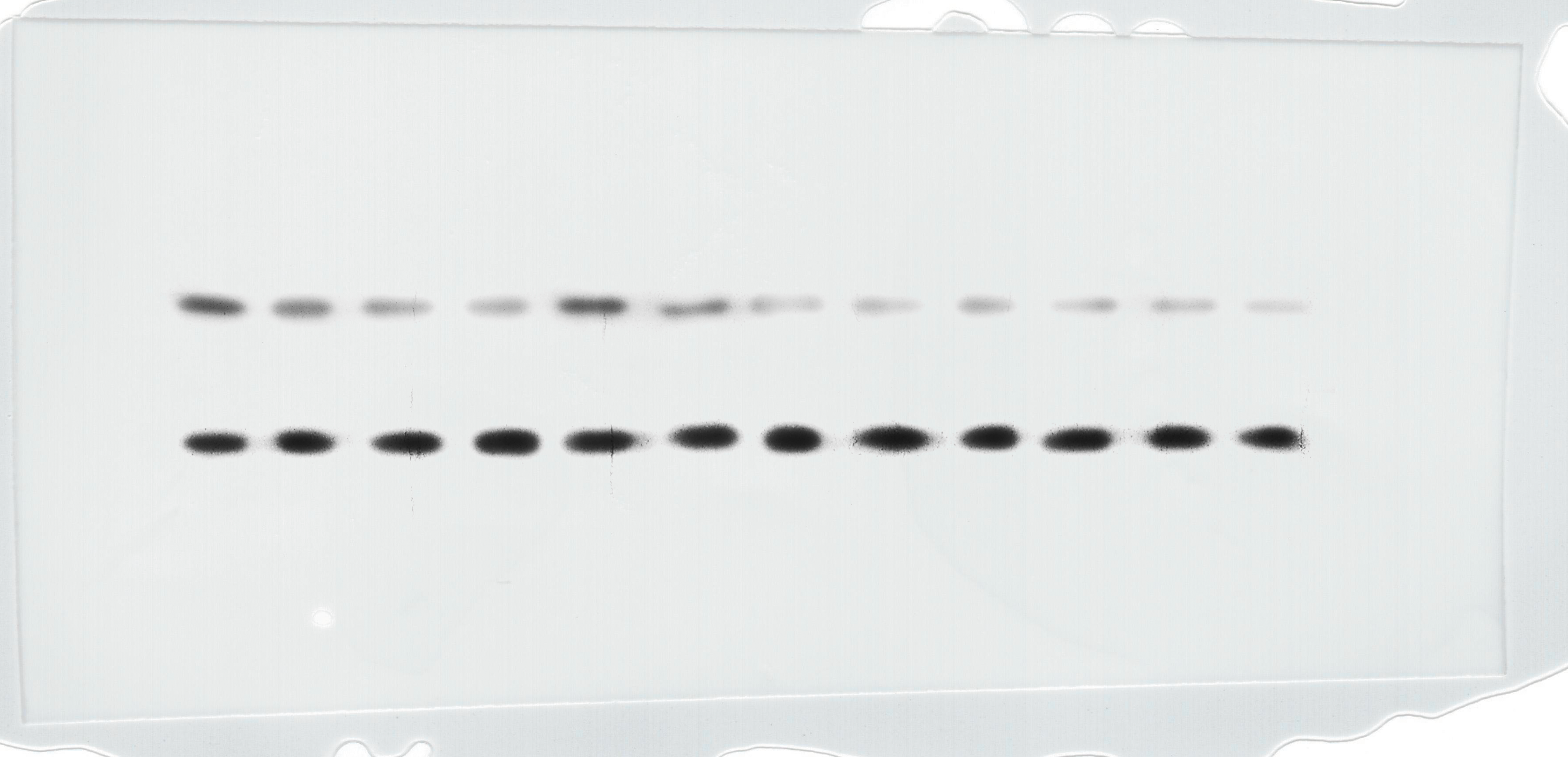

Supplement: Supplementary file 2 [file DataSheet_2.zip › Part 2/Western Blot/Part 2-Western Blot.tif]

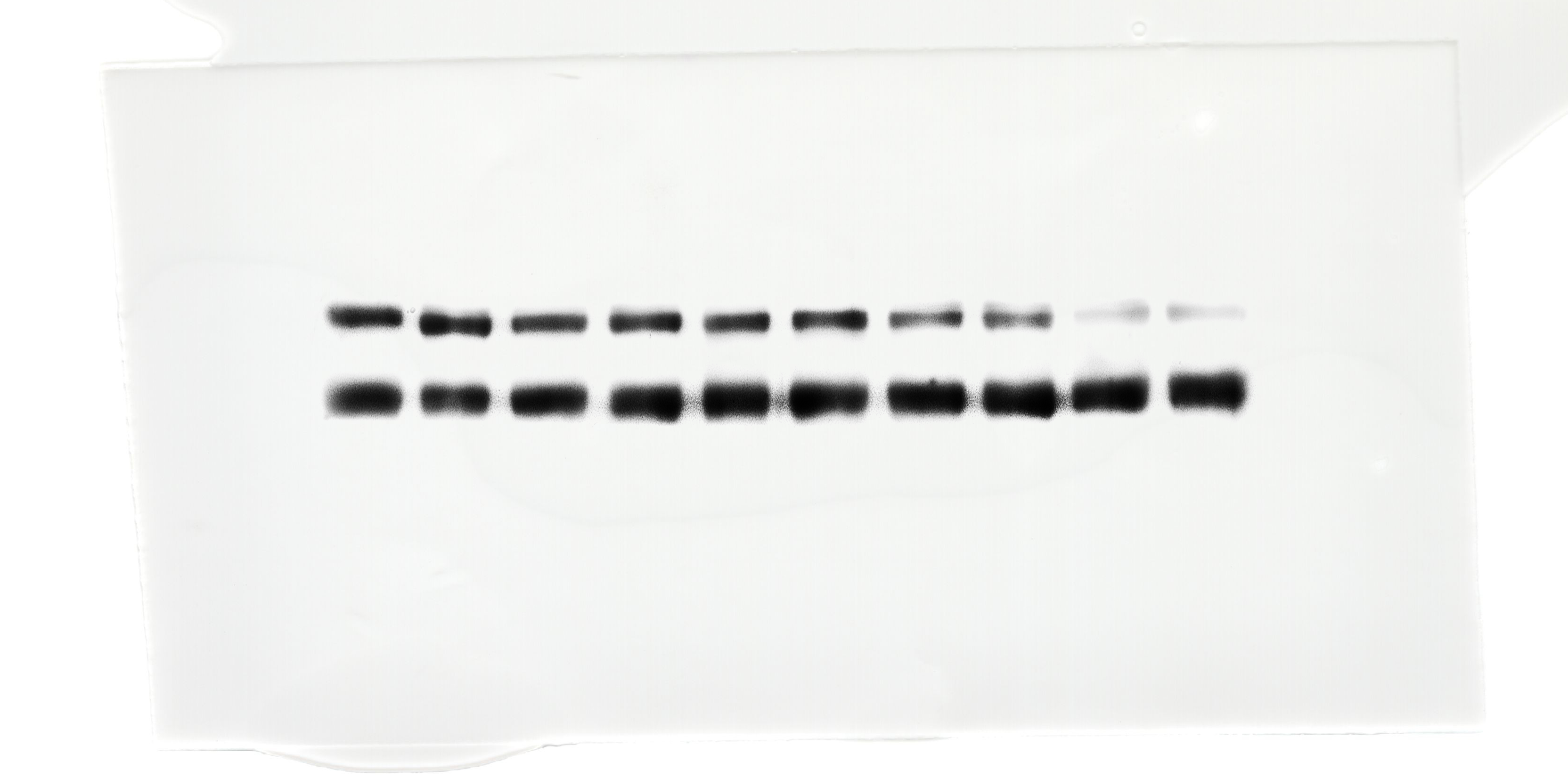

Supplement: Supplementary file 3 [file DataSheet_3.zip › Part 3/Western Blot/Part 3-CX43 Western Blot original membrane.tif]

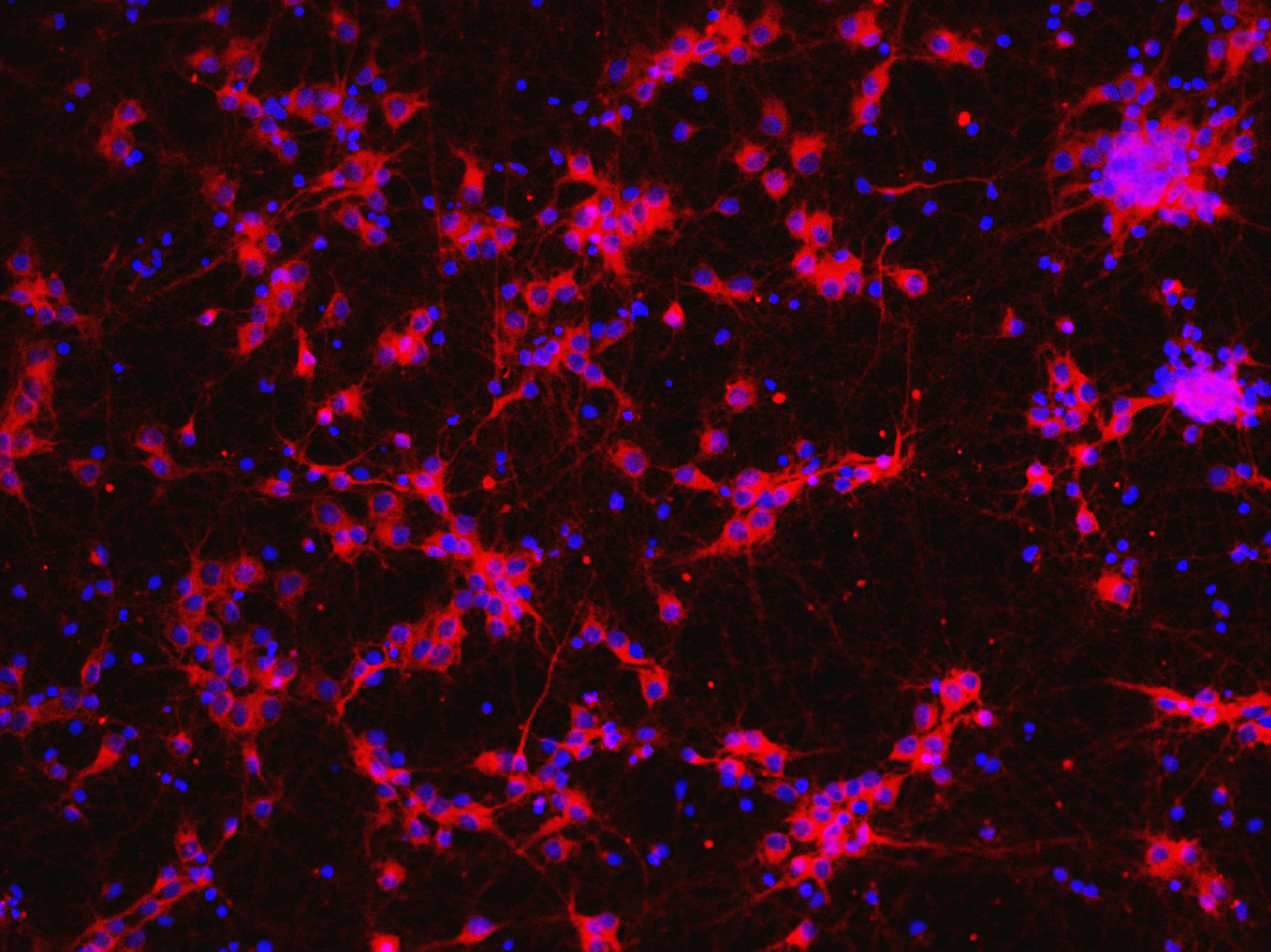

Supplement: Supplementary file 5 [file Image_1.tif]

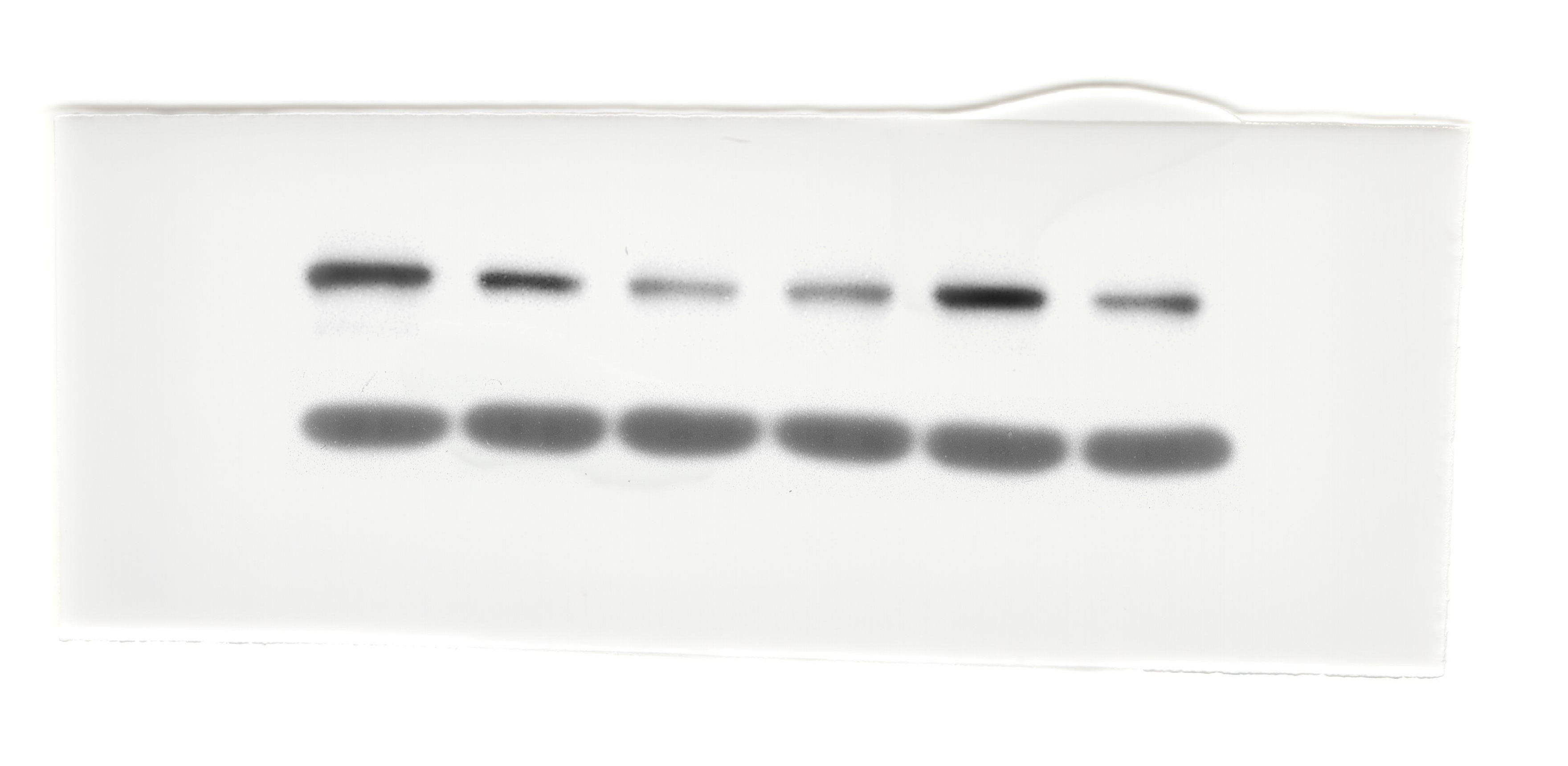

Supplement: Supplementary file 6 [file Image_2.tif]

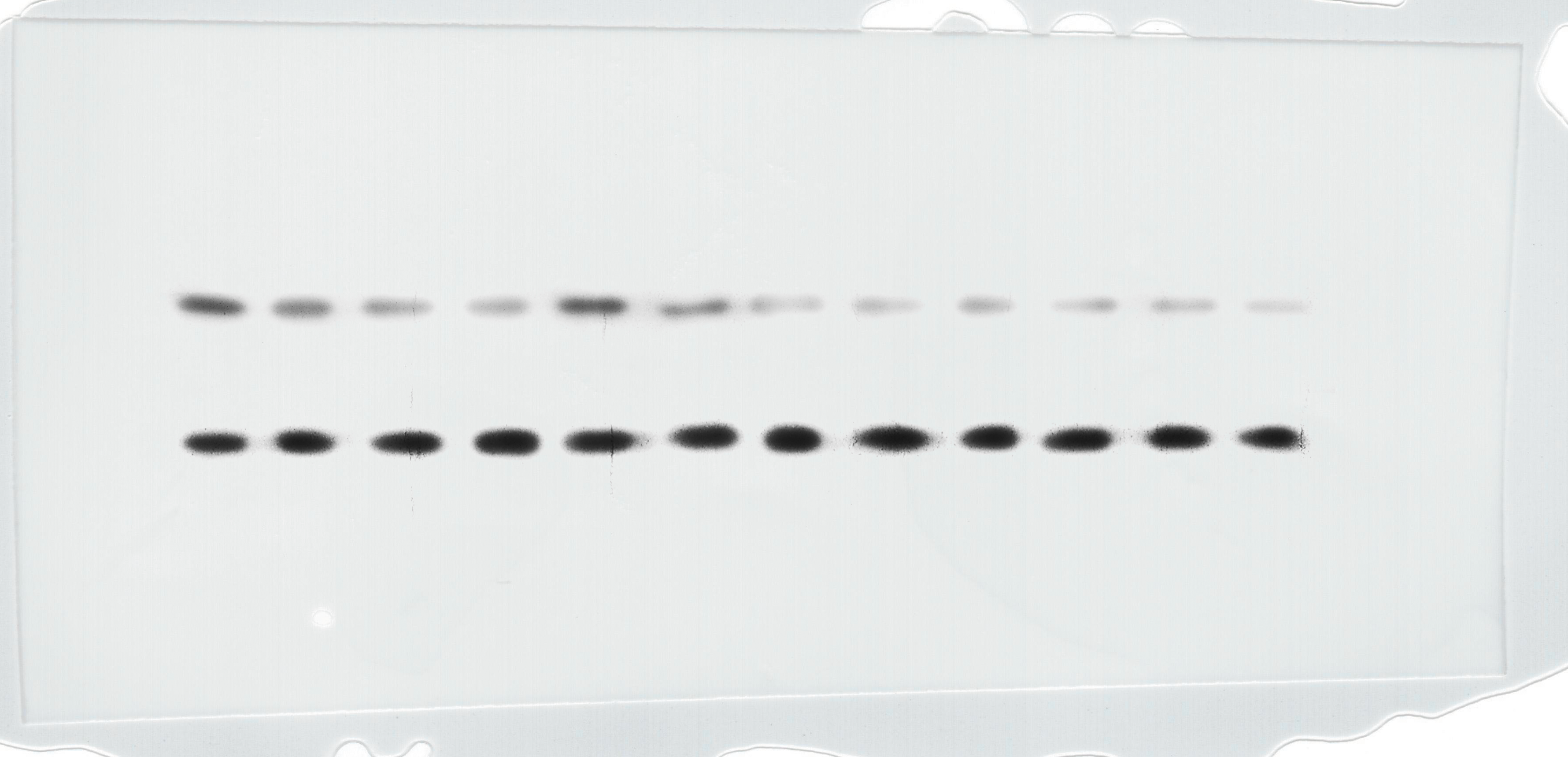

Supplement: Supplementary file 7 [file Image_3.tif]

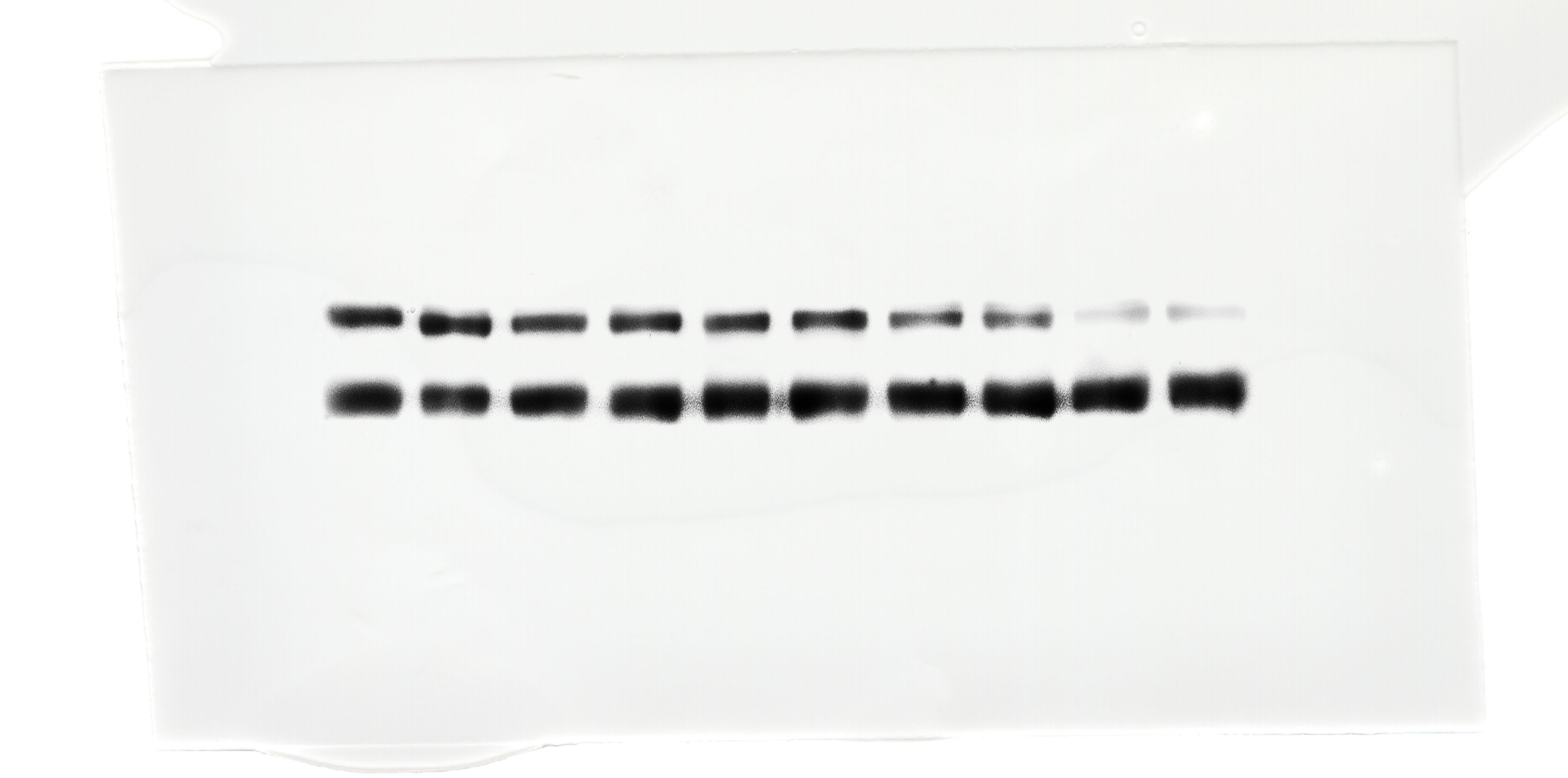

Supplement: Supplementary file 8 [file Image_4.tif]
